# Phylogenetic Analyses of Rotavirus A, B and C Detected on a Porcine Farm in South Africa

**DOI:** 10.3390/v16060934

**Published:** 2024-06-08

**Authors:** Amy Strydom, Neo Segone, Roelof Coertze, Nikita Barron, Muller Strydom, Hester G. O’Neill

**Affiliations:** 1 Department of Microbiology and Biochemistry, University of the Free State, Bloemfontein 9300, South Africa; aimster.strydom@gmail.com (A.S.); neozagonene@gmail.com (N.S.); roelof.coertze@gu.se (R.C.); barronnikita@gmail.com (N.B.); 2 Department of Infectious Diseases, University of Gothenburg, Guldhedsgatan 10, SE-413 46 Göteborg, Sweden; 3 George Animal Hospital, George 6530, South Africa; vet@georgevet.co.za

**Keywords:** porcine rotavirus, rotavirus A, B, C, P-type reassortment, South Africa

## Abstract

Rotaviruses (RVs) are known to infect various avian and mammalian hosts, including swine. The most common RVs associated with infection in pigs are A, B, C and H (RVA-C; RVH). In this study we analysed rotavirus strains circulating on a porcine farm in the Western Cape province of South Africa over a two-year period. Whole genomes were determined by sequencing using Illumina MiSeq without prior genome amplification. Fifteen RVA genomes, one RVB genome and a partial RVC genome were identified. Phylogenetic analyses of the RVA data suggested circulation of one dominant strain (G5-P[6]/P[13]/P[23]-I5-R1-C1-M1-A8-N1-T7-E1-H1), typical of South African porcine strains, although not closely related to previously detected South African porcine strains. Reassortment with three VP4-encoding P genotypes was detected. The study also reports the first complete RVB genome (G14-P[5]-I13-R4-C4-M4-A10-T4-E4-H7) from Africa. The partial RVC (G6-P[5]-IX-R1-C1-MX-A9-N6-T6-EX-H7) strain also grouped with porcine strains. The study shows the continued circulation of an RVA strain, with a high reassortment rate of the VP4-encoding segment, on the porcine farm. Furthermore, incidents of RVB and RVC on this farm emphasize the complex epidemiology of rotavirus in pigs.

## 1. Introduction

Rotavirus (RV) causes acute gastroenteritis in various mammalian, including humans and livestock, and avian species. Nine species of rotaviruses have been classified based on VP6 variation, namely A–D and F–J, according to the International Committee on Taxonomy of Viruses (ICTV) ([1]; https://talk.ictvonline.org/taxonomy/ (accessed on 9 March 2024)). RVA has by far the biggest public health impact and is therefore the best studied of all RVs. The virus contains a segmented, double-strand RNA genome, consisting of 11 segments and encoding 6 structural proteins and 5/6 non-structural proteins [2]. These proteins have been classified according to genotype: VP7 (G)—VP4 (P)—VP6 (I)—VP1 (R)—VP2 (C)—VP3 (M)—NSP1 (A)—NSP2 (N)—NSP3 (T)—NSP4 (E)—NSP5/6 (H) with 42G, 58P, 32I, 28R, 24C, 24M, 39A, 28N, 28T, 32E and 28H genotypes assigned to RVA (Rotavirus Classification Working Group: (RCWG). Available online: https://rega.kuleuven.be/cev/viralmetagenomics/virus-classification/rcwg (accessed on 9 March 2024)).

Compared to RVA, knowledge of RVB and RVC is limited despite the impact of these viruses on mortality rates and economic loses in the agricultural sector [3,4,5,6,7]. Advancements in next-generation sequencing initiatives have, however, led to proposals for whole-genome classification of RVB and RVC similar to that for RVA. An RVB classification system was first described in 2018, while revised genotyping was proposed in 2023. Currently, 27G, 6P, 13I, 7R, 6C, 5M, 8A, 10N, 6T, 4E, and 7H types have been identified [8,9]. These genotypes are, for the most part, host–specific for porcine, human, bovine, caprine and murine hosts. Similarly, genotypes described for RVC are also host-specific with only a few exceptions. In 2021, 31G, 26P, 13I, 5R, 5C, 5M, 12A, 10N, 9T, 8E and 4H types were described for RVC strains detected in porcine, human, bovine, canine and ferret [10].

Rotaviruses are endemic in pig populations and varying detection rates have been reported [11,12,13,14]. Infected pigs can have clinical or subclinical symptoms with neonatal and suckling piglets worst affected. Rotaviruses A, B, C and H have all been detected in pigs. RVA and RVC are associated with diarrhoea in piglets and weaning animals, whereas RVB has been more associated with older animals [11,14]. Twelve G and 16 P RVA types have been detected in pigs, of which G5P [7] was reported to be the most frequently detected genotype combination [15]. Similarly, 15 G and 16 P RVC types and 27 G and 3 P RVB types have been detected in pigs [9,11]. The probability that porcine populations can act as reservoirs for human infection has been discussed before, and multiple studies have reported evidence of zoonotic transmission and reassortment events of RVA strains [16,17,18,19].

Porcine rotavirus was first identified in 1977 in South Africa in faecal samples using electron microscopy [20]. Between 1992 and 1993, rotavirus A, B, and C were identified in porcine faecal samples throughout South Africa [21,22,23]. This was also the first detection of any non-RVA in Africa [24]. African human RVA strains exhibit a high degree of genotype diversity. Proximity to livestock and the frequency of co-infections with bovine or porcine strains leading to human–animal reassortment events contribute to the diversity [25,26,27]. However, very little is known about the animal RVA strains in Africa and no surveillance is performed in South Africa. Even less is known about RVB and RVC, and it remains to be seen how Africa compares to developed countries. At the beginning of 2018, we were approached to confirm the occurrence of rotavirus on a porcine farm in the Western Cape of South Africa. The first sample we received tested positive for rotavirus, which prompted further sample testing. Since samples were received infrequently, rotavirus prevalence could not be determined; rather, the study aimed to determine genetic variation in rotavirus on the porcine farm over the course of two years.

## 2. Materials and Methods

### 2.1. Sampling and Rotavirus Detection

This animal study was conducted with the approval of the Animal Research Ethics Committee at the University of the Free State (UFS) (UFS-AED2018/0030). One hundred and twenty-one samples were collected on a porcine farm in the Western Cape province of South Africa between January 2018 and February 2020. The farm is a born-to-finish and all in–all out system with a 370-sow unit. At the time of sampling, the average born alive per sow was about 12 piglets. Piglets were weaned at 28 days, moved to a weaner house where they were housed up to 60 days, in their respective groups, after which they were moved to a porker house and stayed there until 84 days, before finally being moved to the grower house until slaughter at 154 days. Samples, both symptomatic (liquid) and asymptomatic (solid), were collected directly from the surface of porcine pens of the farrowing (>28 days) and weaner houses (28–60 days). Total RNA was extracted from the 121 stool samples using Tri-reagent (Sigma Aldrich, St. Louis, MO, USA), and single-stranded RNA was precipitated with 1 M lithium chloride [28]. Extracted RNA was examined by gel electrophoresis on 1% agarose gel and samples with typical rotavirus migration patterns were recorded as positive.

### 2.2. cDNA Synthesis and Next-Generation Sequencing

The dsRNA of samples with rotavirus profiles were treated with 9 U of DNase I (Sigma Aldrich, St. Louis, MO, USA). An anchor primer was annealed to the dsRNA before sequencing in order to obtain full-length gene segments as previously described [28]. Complementary DNA was synthesized with the Maxima H Minus Double Stranded cDNA kit (Thermo Fisher Scientific, Waltham, MA, USA) using random hexamers. Minor modifications to the manufacturer’s instructions included denaturing of the dsRNA at 95 °C for 5 min and first-strand synthesis for two hours at 50 °C [29]. Purified cDNA was submitted for sequencing at the UFS Next-Generation Sequencing Unit (UFS-NGS, Bloemfontein, South Africa). To perform whole-genome sequencing, an Illumina Miseq sequencer (Illumina, Inc., San Diego, CA, USA) was used. Sequencing was performed using a Miseq Reagent kit V2 (500 cycles) with 251 × 2 paired end reads.

### 2.3. Data Assembly and Analysis

All paired-end reads were screened for poor-quality nucleotides, which were removed using Trimmomatic [30]. Sequencing adapters were clipped and reads shorter than 50 bp were discarded. Additionally, a sliding window of four base pairs was used to remove flanking nucleotides if the average quality score dropped below 20. The overall quality of the reads was assessed before and after trimming using FastQC. De novo assembly of the high-quality paired-end reads was carried out using SPAdes and its default parameters [31]. The identities of the assembled contigs were determined by comparing them to the nucleotide BLAST database, specifying rotavirus A, B, and C. Database matches were used to select possible reference sequences to perform reference mapping for more reliable consensus sequences (Table S1). Reference mapping against the reference sequences was performed locally using Bowtie 2 [32]. Strict mismatch parameters were selected to ensure high-accuracy reference mapping. Calculation of mapping coverage and extraction of consensus sequences was performed using Samtools [33].

Consensus sequences were analysed in BLASTn and RVA genotypes were identified with the Virus Pathogen Database and Analysis Resource (ViPR) [34]. Reference sequences for RVA, B, and C were obtained from GenBank for phylogenetic analyses. The sequences of each segment were aligned with the appropriate reference sequences using MUSCLE in MEGA X [35]. Maximum-likelihood trees were generated using IQtree using the optimal substitution model and ultrafast bootstrap approximation approach [36,37]. Nucleotide distance matrixes were calculated using the p-distance algorithm in MEGA X. Genotypes for RVB and RVC were assigned based on the most recent classification [8,10]. The nucleotide sequence data presented have been deposited in GenBank under the following accession numbers PP669365-PP669534 (RVA), PP669283-PP669293 (RVB) and PP669294-PP669301 (RVC).

## 3. Results

### 3.1. Sequencing of Rotavirus-Positive Samples

Electrophoretic analysis of the extracted dsRNA suggested the presence of rotavirus in 16 of the 121 samples collected (Table 1). All positive samples detected in the farrowing and weaner houses were diarrhetic and detected during each of the five sampling dates. These 16 samples were subjected to next-generation sequencing (NGS), which revealed that 15 samples contained RVA strains. Two of the 15 samples had a co-infection with RVC (UFS-BOC009 and UFS-BOC035) and one with RVB (UFS-BOC124) (Table 1). The remaining sample contained rotavirus B (UFS-BOC050). The co-infected samples were detected during separate sampling trips, indicating circulation of different RV groups over time. The RVB sample detected in December 2019 was also detected in the same pen where RVA was detected (Table 1).

Complete open reading frames (ORFs) were obtained for all RVA genome segments. Average coverage (sequence depth) for the RVA consensus sequences ranged from 107.1 to 6562.86 (Table S2). A complete genome was determined for an RVB strain in sample UFS-BOC050. All genome segments were full length except segments 1 and 10 (99.97% and 88.3%, respectively). Average coverage for the RVB segments ranged from 1932.9 to 4688.0. Although all the segments were detected for an RVB strain in UFS-BOC124, with genome segment lengths ranging from 66.3% to 99.9%, a very low number of sequencing reads (average coverage ranged between 4.5 to 11.1) was obtained (Table S2). Sample UFS-BOC009 contained an RVA strain as well as an RVC strain. The average coverage for the RVA strain ranged from 886.7 to 3687.9, whereas the RVC coverage ranged from 31.8 to 131.3. RVC was also identified in sample UFS-BOC035, but similarly to the RVB strain in UFS-BOC124, the average coverage for the RVC strain in UFS-BOC035 was low (5.1 to 18.9) (Table S2).

### 3.2. Genotyping and Phylogenetic Analyses

#### 3.2.1. Rotavirus A

**Figure 1:**
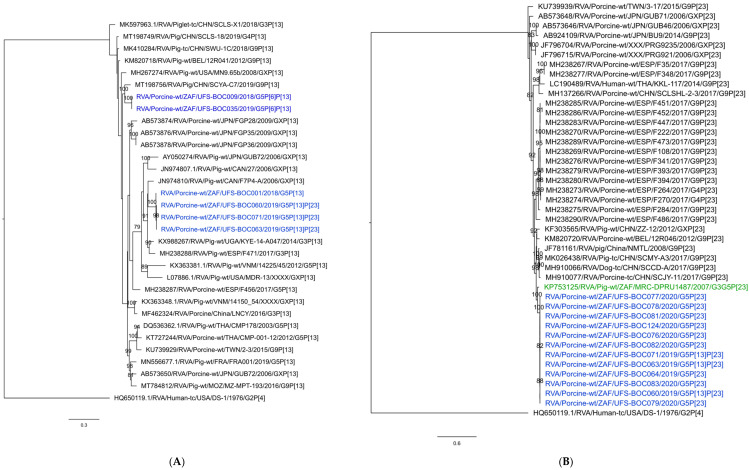

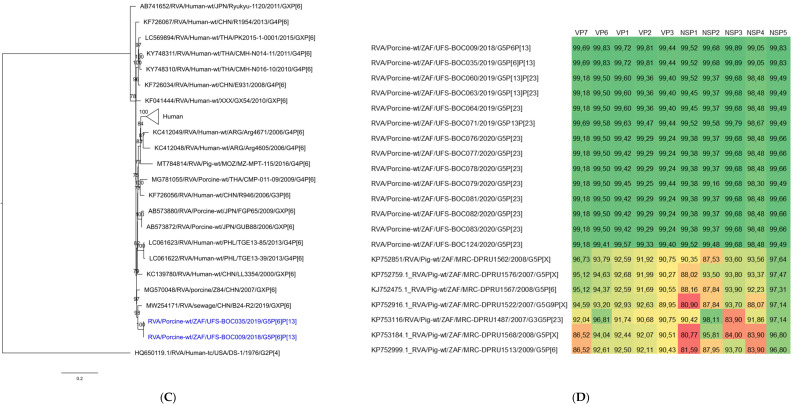
Rotavirus A phylogenetic analyses. (**A**): VP4 (P[13]), (**B**): VP4 (P[23]), (**C**): VP4 (P[6]); (**D**): comparison of BOC001 with other study strains and other South African strains. The heatmap indicates the similarity between the study strains (green) and closely related study strains (yellow to red). The South African study strains in the phylogenetic trees are indicated in blue and previously described South African strains are indicated in green. Each gene was compared with sequences available in GenBank and nucleotide alignments were constructed using the MUSCLE algorithm in MEGA X [35]. Phylogenetic trees were generated using IQtree implementing the maximum-likelihood method with ModelFinder, and the trees were statistically supported using 1000 ultrafast bootstrap runs. For P[23] and P[6], K3Pu + F + I + G4 was used, and for P[13], GTR + F + I + G4 was used. The trees are drawn to scale, with branch lengths in the same units as those of the evolutionary distances used to infer the phylogenetic tree.

The rotavirus A strains were identified as G5-I5-R1-C1-M1-A8-N1-T7-E1-H1 in combination with P[6], P[13] or P[23] (Table 2). All the sequences for each segment, apart from those encoding VP4, are nearly identical to each other (Figure 1 and Figure S1). The closest relatives to VP7, VP6, VP1, VP3, NSP1, NSP2, NSP3 and NSP4 encoding genome segments are all derived from South African RVA strains detected in porcine samples (Figure 1). These strains also grouped together in the phylogenetic trees, and in most instances with previously described South African strains (Figure S1). The exception was the NSP5-encoding sequences determined in this study, which grouped separately from previously described South African strains with strains from non-South African countries. The strains were, however, still closely related to the previously described South African porcine strains with nucleotide identities ranging from 96.63% to 99.83% (Figure 1; Table S3).

The variation in the VP4-encoding genotypes detected on the farm is an interesting observation. Two different P[13] (a and b) sequences were detected during the study. The first sample collected in January 2018 contained a P[13]^a^ genotype (UFS-BOC001). A highly similar P[13]^a^ was detected almost two years later in December 2019 in four samples sourced from the same pen (Table 2; Figure 1). These samples had co-infections with P[23]. The P[13]^a^ sequences had approximate 99.5% nucleotide identity with those detected in January 2018 (Table S3). During December 2018 and February 2019, another two P[13]^b^ strains were detected on the farm with co-infections with P[6] (Table 2). However, these sequences grouped separately from the P[13]^a^ sequences in the phylogenetic tree and only shared an approximate 83.5% nucleotide identity with these strains (Figure 1; Table S3). Interestingly, the closest relative to the P[13]^a^ sequences was from Canada—RVA/Pig-wt/CAN/F7P4-A/2006/GXP[13], with only 90.12% nucleotide identity. Similarly, the closest relative to the P[13]^b^ sequences, RVA/Pig-wt/CHN/SCYA-C7/2019/G9P[13], was from China, with 90.5% nucleotide identity (Table S3).

The P[6] sequences detected in December 2018 and February 2019 (UFS-BOC009 and UFS-BOC035) were identical and clustered in a group with both human and porcine strains from Asia (Figure 1C). The closest relative was a strain from China: RVA/sewage/CHN/B24-R2/2019/GXP[6], with 95.5% nucleotide identity (Table S3). The two P[6] sequences shared only 91.4% nucleotide identity with a P[6]-containing porcine strain from Mozambique (RVA/Pig-wt/MOZ/MZ-MPT-115/2016/G4P[6]), approximately 89% nucleotide identity with porcine strains from South Africa, and approximately 91% nucleotide identity with South African human strains (Table S3).

Twelve P[23] sequences were detected in December 2019 and February 2020 and were all identical. The closest relative was another South African porcine strain, RVA/Pig-wt/ZAF/MRC-DPRU1487/2007/G3G5P[23], with a nucleotide identity of 95.19%.

#### 3.2.2. Rotavirus B

**Figure 2:**
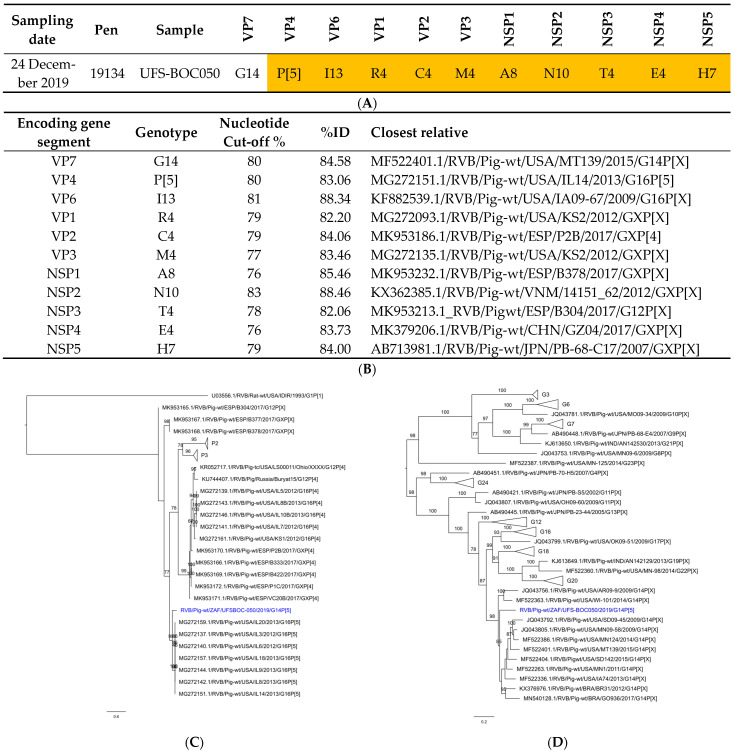
Rotavirus B phylogenetic analyses. (**A**): Genome constellation of UFS-BOC050, (**B**): nucleotide identities of the closest relatives of RVB genes, (**C**,**D**): phylogenetic trees based on the RVB VP4 (**C**) and VP7 (**D**) genes. The South African study strain in the phylogenetic tree is indicated in blue. The sequence was compared with sequences available in GenBank and nucleotide alignments were constructed using the MUSCLE algorithm in MEGA X [35]. The phylogenetic tree was generated using IQtree implementing the maximum-likelihood method with ModelFinder (VP4: TIM3 + F + I + G4; VP7: GTR + F + I + G4) and statistically supported using 1000 ultrafast bootstrap runs. The trees are drawn to scale, with branch lengths in the same units as those of the evolutionary distances used to infer the phylogenetic tree.

Due to the low sequence coverage obtained for UFS-BOC124 only UFS-BOC050 was genotyped. The genome constellation was identified as G14-P[5]-I13-R4-C4-M4-A8-T4-E4-H7 (RVB/Pig-wt/ZAF/UFS-BOC050/2019/G14P[5]) using distance matrices and phylogenetic trees for each segment (Figure 2 and Figure S2; Table S4). The pairwise identity of the closest relatives fell within the ranges for the segments as described in 2018 and updated in 2023 (Figure 2) [8,9]. These genotypes are typically associated with RVB detected in porcine samples, and the closest relatives were all of porcine origin. Segments encoding for VP7, VP4, VP6, VP1 and VP3 were all related to strains from the USA [8,9] detected between 2009 and 2015. Segments encoding for VP2, NSP1 and NSP3 were related to Spanish strains [38], and the remaining segments (NSP2, 4 and 5) were related to Asian strains [16]. The nucleotide identities ranged between 82.1% and 88.5%, indicating that the South African strain is diverse from the previously sequenced RVB strains.

#### 3.2.3. Rotavirus C

**Figure 3:**
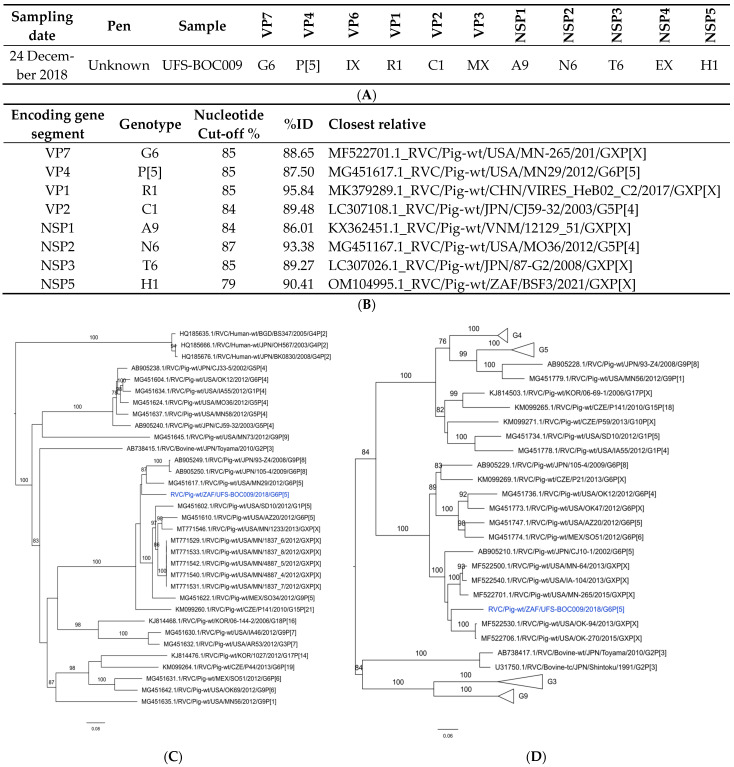
Rotavirus C phylogenetic analyses. (**A**): Genome constellation of UFS-BOC009, (**B**): nucleotide identities of the closest relatives of the RVC genes, (**C**,**D**): phylogenetic trees based on the RVC VP4 (**C**) and VP7 (**D**) genes. The South African study strain in the phylogenetic tree is indicated in blue. The sequence was compared with sequences available in GenBank and nucleotide alignments were constructed using the MUSCLE algorithm in MEGA X [35]. The phylogenetic tree was generated using IQtree implementing the maximum-likelihood method, with ModelFinder (VP7: TIM2 + F + I + G4; VP4: GTR + F + G4) and statistically supported using 1000 ultrafast bootstrap runs. The trees are drawn to scale, with branch lengths in the same units as those of the evolutionary distances used to infer the phylogenetic tree.

Similarly to RVB, the sequence data obtained for UFS-BOC035 were deemed insufficient for genotyping. In addition, the average coverage for VP6-, VP3- and NSP4-encoding sequences of UFS-BOC009 ranged between 31 and 43 and was therefore also excluded from further analysis (Table S2). The partial genome constellation of the RVC strain (RVC/Pig-wt/ZAF/UFS-BOC009/2018/G6P[5]) was identified as G6-P[5]-IX-R1-C1-MX-A9-N6-T6-EX-H7 (Figure 3 and Figure S3; Table S5). The nucleotide identities of the remaining segments were in range with the most recently described cut-off levels [10]. The closest relatives to the study strain segments were all detected in pigs from Asia and the USA. The only exception was NSP5 (nucleotide identity: 90.41%), which was related to a South African strain (RVC/Pig-wt/ZAF/BSF3/2021/GXP[X]) detected in the oral virome of a pig [39]. The oral virome study was conducted in KwaZulu Natal province in South Africa in 2021 and two partial RVC strains were identified. However, most of the sequences were too short to include in the phylogenetic analyses of the present study, since only 19–50% of the sequences were determined [39]. Significant diversity from known RVC strains was again observed, with only the VP1-encoding gene of the study strain exhibiting a comparatively high nucleotide identity of 95.84% with RVC/Pig-wt/CHN/VIRES_HeB02_C/2017/GXP[X].

## 4. Discussion

The characterisation and genetic surveillance of porcine rotavirus strains are important on two fronts: firstly, to determine the presence and diversity of the virus causing economic loses in the pork industry for risk analyses and management strategies; and secondly, to understand the influence that porcine rotaviruses might have on the genetic diversity of human strains and their impact on public health. This study describes the genetic diversity of rotavirus strains detected over a two-year period on a porcine farm in the Western Cape province of South Africa. Study limitations include low sample numbers, inconsistent sample sizes, infrequent sampling dates and the fact that samples could only be linked to a pen and not a specific animal. We therefore did not set out to systematically analyse the prevalence of rotavirus, but rather determine the genetic variance of the rotavirus population.

The ability of pigs to harbour species A, B, C, and H (not detected in this study) is well known [11], and the simultaneous detection of multiple species on a farm is not unique either [14]. In a study conducted in Eastern Australia, species A, B, and C were detected in piggeries as mono-infections, but also co-infections. The study reported a higher prevalence for RVA in young pigs (piglets and weaners) whereas RVB and RVC were also detected in older animals (>11 weeks) [14]. Since most of the samples analysed in the current study were obtained from pens housing >28-day-old piglets and weaners, it could explain the higher detection rate for RVA compared to RVB and RVC.

The similarity and consistency between the sequences of the VP7-encoding segment and those of the backbones indicate that there was one dominant RVA strain circulating on the farm for at least two years. Phylogenetic and distance matrix analysis suggested that this porcine RVA strain was similar but not closely related to other South African porcine strains, suggesting the detection of a new porcine strain. The NSP5-encoding segment was the only segment that grouped with non-South African strains, which indicates a possible historical reassortment event for genome segment 11.

The variation in the VP4-encoding segments is of great interest. The origin of the various P types detected in the study is unclear and could indicate that additional RVA strains were present on the farm during the two-year study period. Inconsistency in sample size and infrequent sampling are possible factors that could have contributed to the non-detection of such strains. In the mature virion, the VP4 spike protein fits into a pocket created by VP7 and VP6, and multiple protein–protein interactions ensure the stability of the spike protein [40]. Therefore, the variation in VP4 in the presence of the same VP7 and VP6 proteins warrants further investigation to understand the ability of the porcine strain to harbour different P types.

The P types detected in this study are often reported in porcine RVA studies [11]. It is important to note, though, that in Africa the P[6] genotype is also frequently detected in humans [24,41]. Human and porcine P[6] sequences often cluster together during phylogenetic analysis [41], as was also the case for the P[6] detected in this study, which highlights the zoonotic potential of this genotype. The P[13] sequences identified in the study were not only diverse from each other but also from previously described African P[13] sequences from Uganda and Mozambique [42,43]. Genetic diversity among P[13] sequences has previously been reported for strains detected in piglets in the USA [44]. The only P type detected in the study that was relatively closely related to a South African strain was the P[23], with 95% identity to RVA/Pig-wt/ZAF/MRC-DPRU1487/2007/G3G5P[23]. The P[23] genotype was first detected in December 2019. The P[13]a sequence, first detected in January 2018, was also detected in December 2019, but by February 2020, only the P[23] genotype was detected. It is possible that the P[23] sequences were only introduced to the farm in 2019 and outcompeted the other P genotypes. However, only 12 samples were collected during 2018, and it is therefore possible that an earlier P[23] introduction could have been missed.

This study reports the first full-length genome sequence for a porcine RVB strain from Africa. RVB/Pig-wt/ZAF/UFS-BOC050/2019/G14P[5] was detected in the same pen with various P[13]- and P[23]-containing RVA strains in December 2019, whereas traces of RVB was detected in the RVA/Porcine-wt/ZAF/UFS-BOC124/2020/G5P[23]-containing sample collected two months later, in February 2020. The partial RVC/Pig-wt/ZAF/UFS-BOC009/2018/G6P[5] was also co-detected in a sample containing RVA/Porcine-wt/ZAF/UFS-BOC009/2018/G5P[6]P[13]b during December 2018. Traces of RVC were detected two months later during the February 2019 sampling collection. The low number of reads for the RVB and RVC sequences in samples UFS-BOC124 and UFS-BOC035 could be due to ineffective virus replication.

However, since the traces of both these RVB and RVC strains were detected within two months of RVB/Pig-wt/ZAF/UFS-BOC050/2019/G14P[5] and RVC/Pig-wt/ZAF/UFS-BOC009/2018/G6P[5], it is possible that these infections were clearing. The low nucleotide identities to closest relatives observed for most of the RVB and RVC genome sequences emphasises the lack of sequence data for these strains not only from Africa but also globally.

## 5. Conclusions

The detection of three rotavirus species (A, B, and C) during a two-year period on a porcine farm in the Western Cape province of South Africa highlights the complex epidemiology of rotavirus in porcine populations. The phylogenetic analyses revealed that the RVB and RVC sequences represent unknown strains and will contribute to the little genetic information available for these groups.

## Figures and Tables

**Table 1:** Detection of rotavirus A, B, and C in porcine samples.

Collection Date	Samples Collected	Positive Samples	Age of Positive Piglets	Sample	Rotavirus
10 January 2018	1	1	>28 days ^#^	UFS-BOC001	RVA
24 December 2018	11	1	>28 days ^#^	UFS-BOC009	RVA
					RVC
19 February 2019	25	1	>28 days ^#^	UFS-BOC035	RVA
					RVC *
24 December 2019	34	5	5 days	UFS-BOC050	RVB
				UFS-BOC060	RVA
				UFS-BOC063	RVA
				UFS-BOC064	RVA
				UFS-BOC071	RVA
20 February 2020	50	8	28 days	UFS-BOC076	RVA
				UFS-BOC077	RVA
				UFS-BOC078	RVA
			28 days	UFS-BOC079	RVA
			27 days	UFS-BOC081	RVA
				UFS-BOC082	RVA
				UFS-BOC083	RVA
			30 days	UFS-BOC124	RVA
					RVB *

^#^ Exact date-of-birth unknown, but samples obtained from farrowing houses. * Insufficient number of reads to genotype (Table S2).

**Table 2:** Genome constellations of South African porcine rotavirus A strains.

Collection Date	Pen	Strain	VP7	VP4	VP6	VP1	VP2	VP3	NSP1	NSP2	NSP3	NSP4	NSP5
10 January 2018	unknown	UFS-BOC001	G5	P[13]^a^	I5	R1	C1	M1	A8	N1	T7	E1	H1
24 December 2018	unknown	UFS-BOC009	G5	P[6]P[13]^b^	I5	R1	C1	M1	A8	N1	T7	E1	H1
19 February 2019	unknown	UFS-BOC035	G5	P[6]P[13]^b^	I5	R1	C1	M1	A8	N1	T7	E1	H1
24 December 2019	19134	UFS-BOC060	G5	P[13]^a^P[23]	I5	R1	C1	M1	A8	N1	T7	E1	H1
		UFS-BOC063	G5	P[13]^a^P[23]	I5	R1	C1	M1	A8	N1	T7	E1	H1
		UFS-BOC064	G5	P[23]	I5	R1	C1	M1	A8	N1	T7	E1	H1
		UFS-BOC071	G5	P[13]^a^P[23]	I5	R1	C1	M1	A8	N1	T7	E1	H1
20 February2020	18202	UFS-BOC076	G5	P[23]	I5	R1	C1	M1	A8	N1	T7	E1	H1
		UFS-BOC077	G5	P[23]	I5	R1	C1	M1	A8	N1	T7	E1	H1
		UFS-B0C078	G5	P[23]	I5	R1	C1	M1	A8	N1	T7	E1	H1
	18212	UFS-BOC079	G5	P[23]	I5	R1	C1	M1	A8	N1	T7	E1	H1
	18119	UFS-BOC081	G5	P[23]	I5	R1	C1	M1	A8	N1	T7	E1	H1
		UFS-BOC082	G5	P[23]	I5	R1	C1	M1	A8	N1	T7	E1	H1
		UFS-BOC083	G5	P[23]	I5	R1	C1	M1	A8	N1	T7	E1	H1
	18197	UFS-BOC124	G5	P[23]	I5	R1	C1	M1	A8	N1	T7	E1	H1

^a^ and ^b^ represent two different P[13] sequences.

## Data Availability

The nucleotide sequences generated in this study were submitted to GenBank, and accession numbers PP669365-PP669534 (RVA), PP669283-PP669293 (RVB) and PP669294-PP669301 (RVC) were assigned.

## Supplementary Materials

The following supporting information can be downloaded at https://www.mdpi.com/article/10.3390/v16060934/s1. Figure S1: Rotavirus A phylogenetic analysis; Figure S2: Rotavirus B phylogenetic analysis; Figure S3: Rotavirus C phylogenetic analysis; Table S1: Sequences used for reference mapping; Table S2: Evaluation of genome assembly; Table S3: Rotavirus A distance matrix; Table S4: Rotavirus B distance matrix; Table S5: Rotavirus C distance matrix.
