# Supplementary material for: Phylogenetic Analyses of Rotavirus A, B and C Detected on a Porcine Farm in South Africa
Source: Viruses. 2024 Jun 8;16(6):934. doi: 10.3390/v16060934 (PMC11209240; doi:10.3390/v16060934)
Supplement: Supplementary file 1 [file viruses-16-00934-s001.zip › Suppl Docs Strydom et al/Figure S1_RVA trees.pdf]

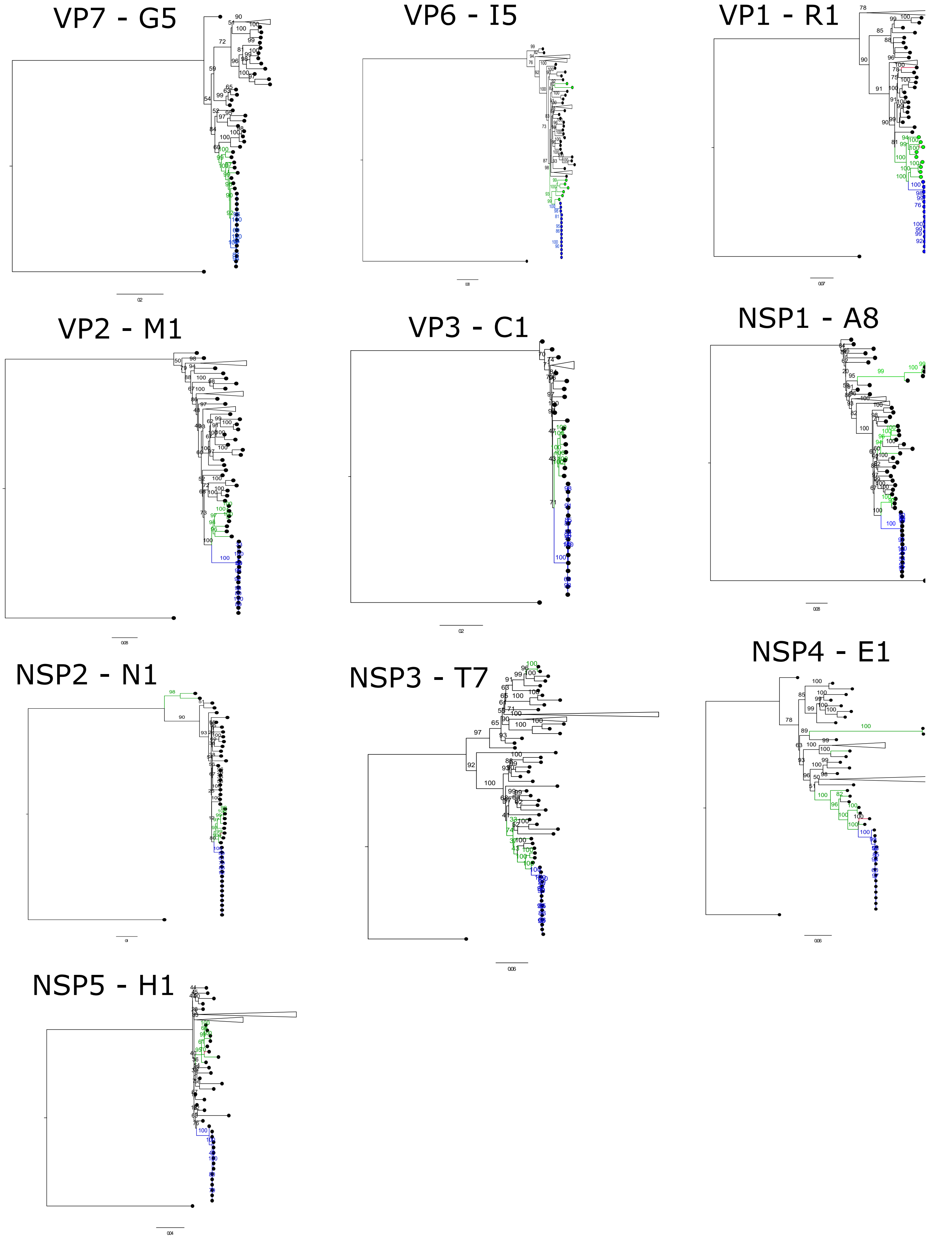
VP7 - G5
VP6 - I5
VP1 - R1
VP2 - M1
VP3 - C1
NSP1 - A8
NSP2 - N1
NSP3 - T7
NSP4 - E1
NSP5 - H1

Figure S1. Rotavirus A Phylogenetic Analyses. The South African study strains in the phylogenetic trees are indicated in blue and other South African strains are indicated in green. Each gene was compared with sequences available in GenBank and nucleotide alignments were constructed using the MUSCLE algorithm in the MEGA X [35]. Phylogenetic trees were generated using IQtree implementing the Maximum Likelihood method, with ModelFinder and the trees were statistically supported using 1000 ultrafast bootstrap runs. For VP7: TPM3+F+I+G4, VP1: GTR+F+G4; VP2, VP3, NSP1 and NSP2: GTR+F+I+G4; VP6: TPM2+F+I+G4 VP6; NSP3 and NSP4 TIM3+F+G4; NSP5: HKY+F+I+G4. The trees are drawn to scale, with branch lengths in the same units as those of the evolutionary distances used to infer the phylogenetic tree.
