# Supplementary material for: Phylogenetic Analyses of Rotavirus A, B and C Detected on a Porcine Farm in South Africa
Source: Viruses. 2024 Jun 8;16(6):934. doi: 10.3390/v16060934 (PMC11209240; doi:10.3390/v16060934)
Supplement: Supplementary file 1 [file viruses-16-00934-s001.zip › Suppl Docs Strydom et al/Figure S2_RVBtrees.pdf]

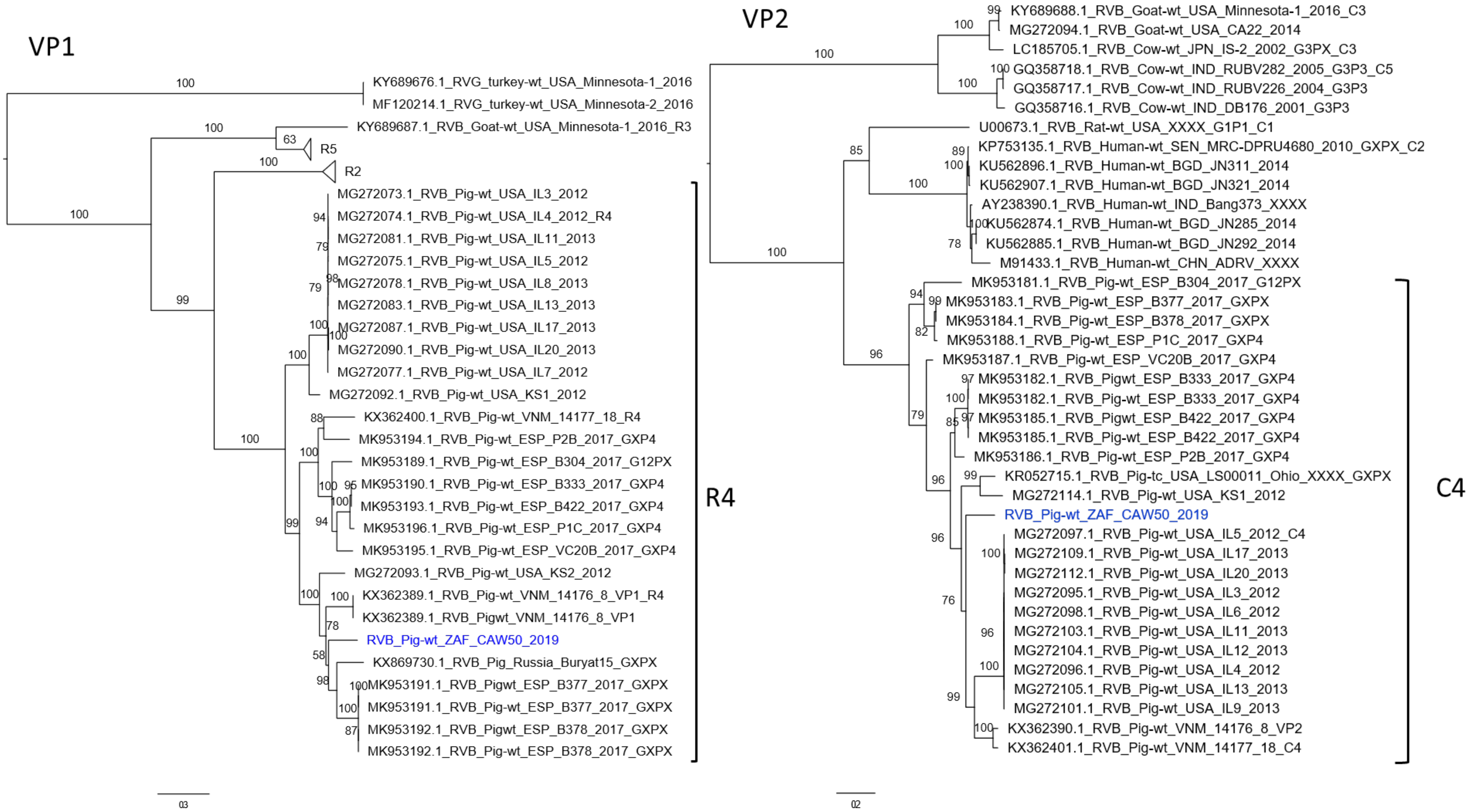
VP1
KY689676.1_RVG_turkey-wt_USA_Minnesota-1_2016
MF120214.1_RVG_turkey-wt_USA_Minnesota-2_2016
KY689687.1_RVB_Goat-wt_USA_Minnesota-1_2016_R3
R5
R2
MG272073.1_RVB_Pig-wt_USA_IL3_2012
MG272074.1_RVB_Pig-wt_USA_IL4_2012_R4
MG272081.1_RVB_Pig-wt_USA_IL11_2013
MG272075.1_RVB_Pig-wt_USA_IL5_2012
MG272078.1_RVB_Pig-wt_USA_IL8_2013
MG272083.1_RVB_Pig-wt_USA_IL13_2013
MG272087.1_RVB_Pig-wt_USA_IL17_2013
MG272090.1_RVB_Pig-wt_USA_IL20_2013
MG272077.1_RVB_Pig-wt_USA_IL7_2012
MG272092.1_RVB_Pig-wt_USA_KS1_2012
KX362400.1_RVB_Pig-wt_VNM_14177_18_R4
MK953194.1_RVB_Pig-wt_ESP_P2B_2017_GXP4
MK953189.1_RVB_Pig-wt_ESP_B304_2017_G12PX
MK953190.1_RVB_Pig-wt_ESP_B333_2017_GXP4
MK953193.1_RVB_Pig-wt_ESP_B422_2017_GXP4
MK953196.1_RVB_Pig-wt_ESP_P1C_2017_GXP4
MK953195.1_RVB_Pig-wt_ESP_VC20B_2017_GXP4
MG272093.1_RVB_Pig-wt_USA_KS2_2012
KX362389.1_RVB_Pig-wt_VNM_14176_8_VP1_R4
KX362389.1_RVB_Pigwt_VNM_14176_8_VP1
RVB_Pig-wt_ZAF_CAW50_2019
KX869730.1_RVB_Pig_Russia_Buryat15_GXPX
MK953191.1_RVB_Pigwt_ESP_B377_2017_GXPX
MK953191.1_RVB_Pig-wt_ESP_B377_2017_GXPX
MK953192.1_RVB_Pigwt_ESP_B378_2017_GXPX
MK953192.1_RVB_Pig-wt_ESP_B378_2017_GXPX
R4
03
VP2
KY689688.1_RVB_Goat-wt_USA_Minnesota-1_2016_C3
MG272094.1_RVB_Goat-wt_USA_CA22_2014
LC185705.1_RVB_Cow-wt_JPN_IS-2_2002_G3PX_C3
GQ358718.1_RVB_Cow-wt_IND_RUBV282_2005_G3P3_C5
GQ358717.1_RVB_Cow-wt_IND_RUBV226_2004_G3P3
GQ358716.1_RVB_Cow-wt_IND_DB176_2001_G3P3
U00673.1_RVB_Rat-wt_USA_XXXX_G1P1_C1
KP753135.1_RVB_Human-wt_SEN_MRC-DPRU4680_2010_GXPX_C2
KU562896.1_RVB_Human-wt_BGD_JN311_2014
KU562907.1_RVB_Human-wt_BGD_JN321_2014
AY238390.1_RVB_Human-wt_IND_Bang373_XXXX
KU562874.1_RVB_Human-wt_BGD_JN285_2014
KU562885.1_RVB_Human-wt_BGD_JN292_2014
M91433.1_RVB_Human-wt_CHN_ADRV_XXXX
MK953181.1_RVB_Pig-wt_ESP_B304_2017_G12PX
MK953183.1_RVB_Pig-wt_ESP_B377_2017_GXPX
MK953184.1_RVB_Pig-wt_ESP_B378_2017_GXPX
MK953188.1_RVB_Pig-wt_ESP_P1C_2017_GXP4
MK953187.1_RVB_Pig-wt_ESP_VC20B_2017_GXP4
MK953182.1_RVB_Pigwt_ESP_B333_2017_GXP4
MK953182.1_RVB_Pig-wt_ESP_B333_2017_GXP4
MK953185.1_RVB_Pigwt_ESP_B422_2017_GXP4
MK953185.1_RVB_Pig-wt_ESP_B422_2017_GXP4
MK953186.1_RVB_Pig-wt_ESP_P2B_2017_GXP4
KR052715.1_RVB_Pig-tc_USA_LS00011_Ohio_XXXX_GXPX
MG272114.1_RVB_Pig-wt_USA_KS1_2012
RVB_Pig-wt_ZAF_CAW50_2019
MG272097.1_RVB_Pig-wt_USA_IL5_2012_C4
MG272109.1_RVB_Pig-wt_USA_IL17_2013
MG272112.1_RVB_Pig-wt_USA_IL20_2013
MG272095.1_RVB_Pig-wt_USA_IL3_2012
MG272098.1_RVB_Pig-wt_USA_IL6_2012
MG272103.1_RVB_Pig-wt_USA_IL11_2013
MG272104.1_RVB_Pig-wt_USA_IL12_2013
MG272096.1_RVB_Pig-wt_USA_IL4_2012
MG272105.1_RVB_Pig-wt_USA_IL13_2013
MG272101.1_RVB_Pig-wt_USA_IL9_2013
KX362390.1_RVB_Pig-wt_VNM_14176_8_VP2
KX362401.1_RVB_Pig-wt_VNM_14177_18_C4
C4
02

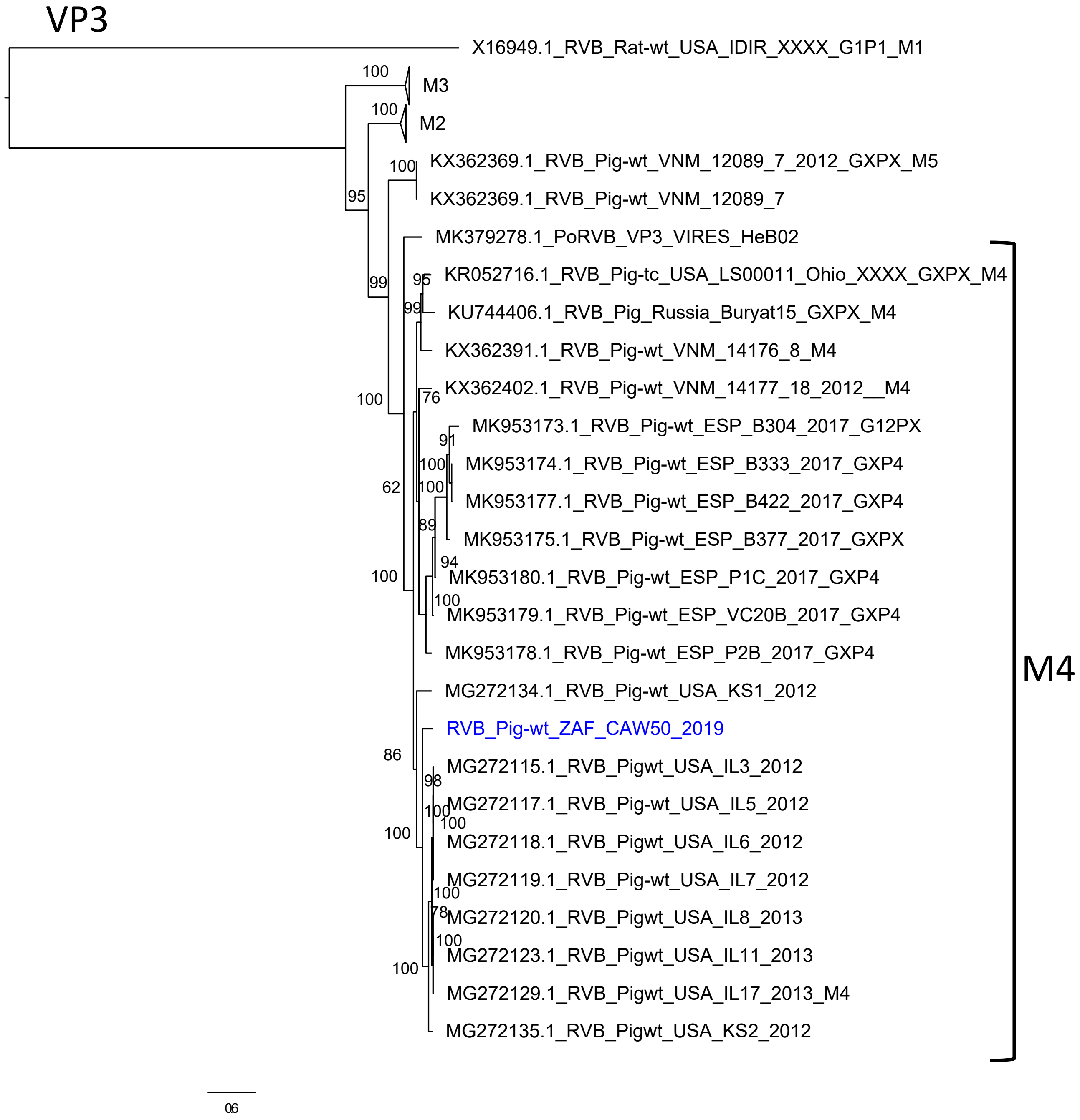
VP3
X16949.1_RVB_Rat-wt_USA_IDIR_XXXX_G1P1_M1
M3
M2
KX362369.1_RVB_Pig-wt_VNM_12089_7_2012_GXPX_M5
KX362369.1_RVB_Pig-wt_VNM_12089_7
MK379278.1_PoRVB_VP3_VIRES_HeB02
KR052716.1_RVB_Pig-tc_USA_LS00011_Ohio_XXXX_GXPX_M4
KU744406.1_RVB_Pig_Russia_Buryat15_GXPX_M4
KX362391.1_RVB_Pig-wt_VNM_14176_8_M4
KX362402.1_RVB_Pig-wt_VNM_14177_18_2012__M4
MK953173.1_RVB_Pig-wt_ESP_B304_2017_G12PX
MK953174.1_RVB_Pig-wt_ESP_B333_2017_GXP4
MK953177.1_RVB_Pig-wt_ESP_B422_2017_GXP4
MK953175.1_RVB_Pig-wt_ESP_B377_2017_GXPX
MK953180.1_RVB_Pig-wt_ESP_P1C_2017_GXP4
MK953179.1_RVB_Pig-wt_ESP_VC20B_2017_GXP4
MK953178.1_RVB_Pig-wt_ESP_P2B_2017_GXP4
MG272134.1_RVB_Pig-wt_USA_KS1_2012
RVB_Pig-wt_ZAF_CAW50_2019
MG272115.1_RVB_Pigwt_USA_IL3_2012
MG272117.1_RVB_Pig-wt_USA_IL5_2012
MG272118.1_RVB_Pigwt_USA_IL6_2012
MG272119.1_RVB_Pig-wt_USA_IL7_2012
MG272120.1_RVB_Pigwt_USA_IL8_2013
MG272123.1_RVB_Pigwt_USA_IL11_2013
MG272129.1_RVB_Pigwt_USA_IL17_2013_M4
MG272135.1_RVB_Pigwt_USA_KS2_2012
M4
06

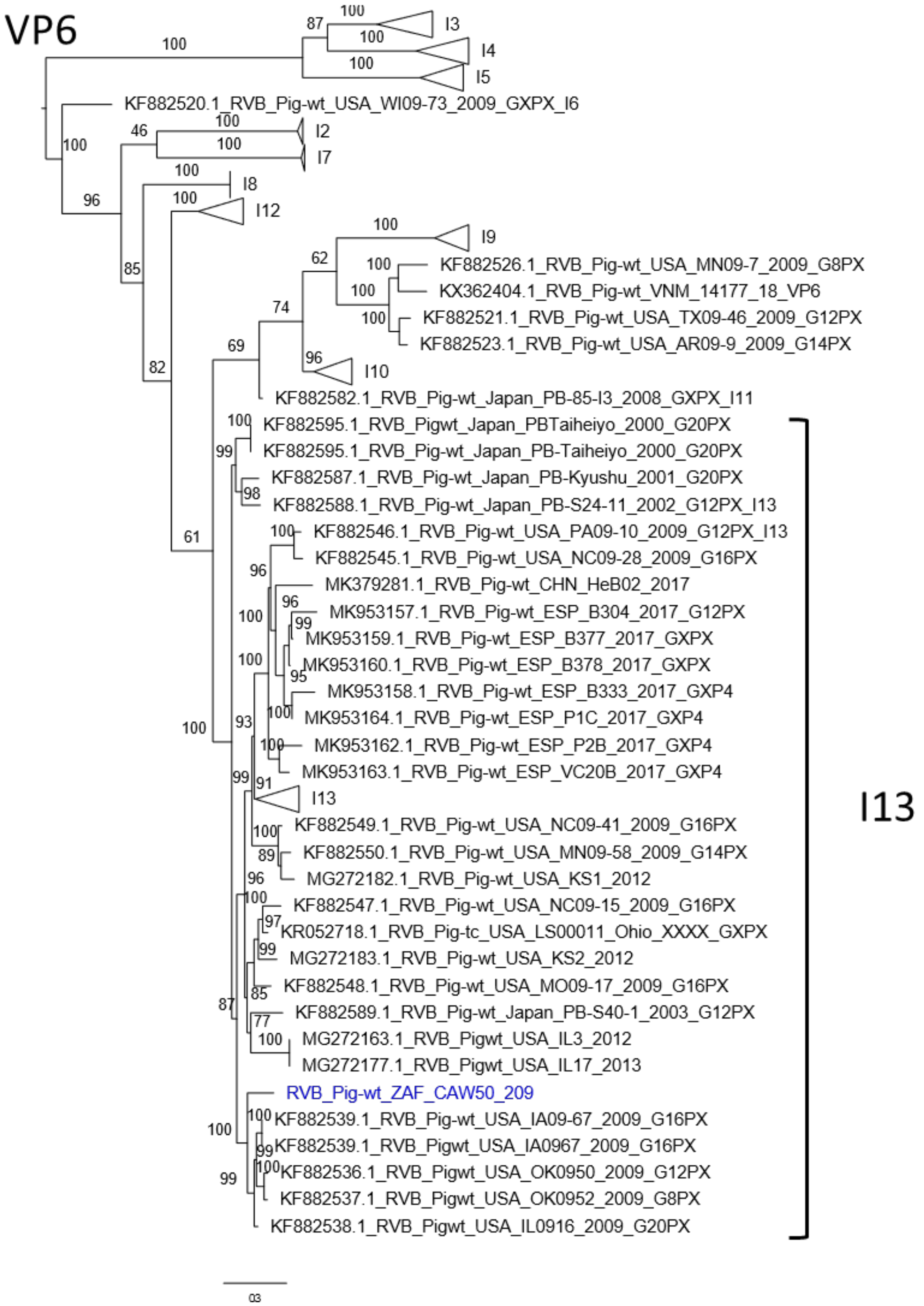
VP6
I3
I4
I5
KF882520.1_RVB_Pig-wt_USA_WI09-73_2009_GXPX_I6
I2
I7
I8
I12
I9
KF882526.1_RVB_Pig-wt_USA_MN09-7_2009_G8PX
KX362404.1_RVB_Pig-wt_VNM_14177_18_VP6
KF882521.1_RVB_Pig-wt_USA_TX09-46_2009_G12PX
KF882523.1_RVB_Pig-wt_USA_AR09-9_2009_G14PX
I10
KF882582.1_RVB_Pig-wt_Japan_PB-85-I3_2008_GXPX_I11
KF882595.1_RVB_Pigwt_Japan_PBTaiheiyo_2000_G20PX
KF882595.1_RVB_Pig-wt_Japan_PB-Taiheiyo_2000_G20PX
KF882587.1_RVB_Pig-wt_Japan_PB-Kyushu_2001_G20PX
KF882588.1_RVB_Pig-wt_Japan_PB-S24-11_2002_G12PX_I13
KF882546.1_RVB_Pig-wt_USA_PA09-10_2009_G12PX_I13
KF882545.1_RVB_Pig-wt_USA_NC09-28_2009_G16PX
MK379281.1_RVB_Pig-wt_CHN_HeB02_2017
MK953157.1_RVB_Pig-wt_ESP_B304_2017_G12PX
MK953159.1_RVB_Pig-wt_ESP_B377_2017_GXPX
MK953160.1_RVB_Pig-wt_ESP_B378_2017_GXPX
MK953158.1_RVB_Pig-wt_ESP_B333_2017_GXP4
MK953164.1_RVB_Pig-wt_ESP_P1C_2017_GXP4
MK953162.1_RVB_Pig-wt_ESP_P2B_2017_GXP4
MK953163.1_RVB_Pig-wt_ESP_VC20B_2017_GXP4
I13
KF882549.1_RVB_Pig-wt_USA_NC09-41_2009_G16PX
KF882550.1_RVB_Pig-wt_USA_MN09-58_2009_G14PX
MG272182.1_RVB_Pig-wt_USA_KS1_2012
KF882547.1_RVB_Pig-wt_USA_NC09-15_2009_G16PX
KR052718.1_RVB_Pig-tc_USA_LS00011_Ohio_XXXX_GXPX
MG272183.1_RVB_Pig-wt_USA_KS2_2012
KF882548.1_RVB_Pig-wt_USA_MO09-17_2009_G16PX
KF882589.1_RVB_Pig-wt_Japan_PB-S40-1_2003_G12PX
MG272163.1_RVB_Pigwt_USA_IL3_2012
MG272177.1_RVB_Pigwt_USA_IL17_2013
RVB_Pig-wt_ZAF_CAW50_209
KF882539.1_RVB_Pig-wt_USA_IA09-67_2009_G16PX
KF882539.1_RVB_Pigwt_USA_IA0967_2009_G16PX
KF882536.1_RVB_Pigwt_USA_OK0950_2009_G12PX
KF882537.1_RVB_Pigwt_USA_OK0952_2009_G8PX
KF882538.1_RVB_Pigwt_USA_IL0916_2009_G20PX
I13
03

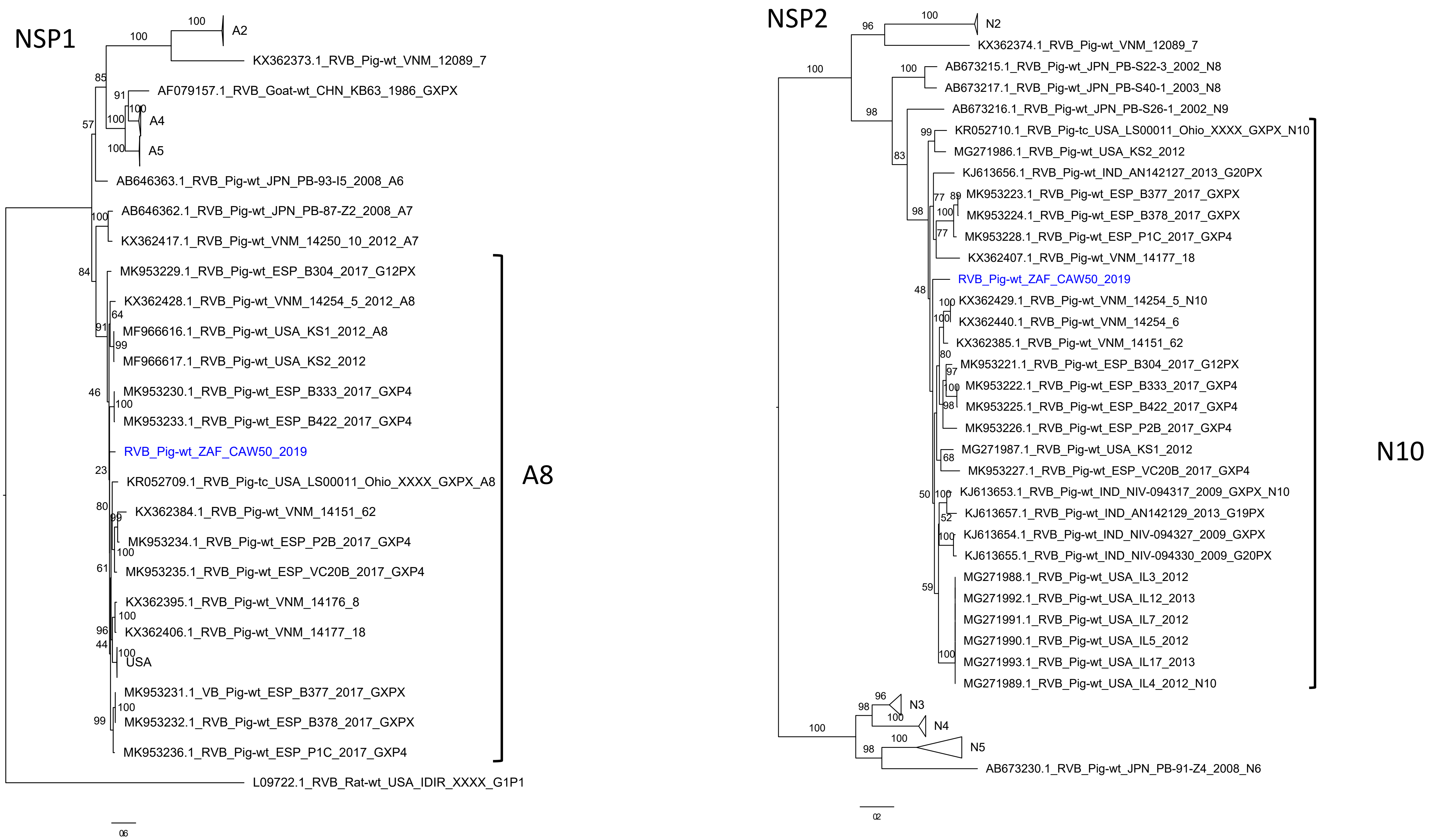
NSP1
A2
KX362373.1_RVB_Pig-wt_VNM_12089_7
AF079157.1_RVB_Goat-wt_CHN_KB63_1986_GXPX
A4
A5
AB646363.1_RVB_Pig-wt_JPN_PB-93-I5_2008_A6
AB646362.1_RVB_Pig-wt_JPN_PB-87-Z2_2008_A7
KX362417.1_RVB_Pig-wt_VNM_14250_10_2012_A7
MK953229.1_RVB_Pig-wt_ESP_B304_2017_G12PX
KX362428.1_RVB_Pig-wt_VNM_14254_5_2012_A8
MF966616.1_RVB_Pig-wt_USA_KS1_2012_A8
MF966617.1_RVB_Pig-wt_USA_KS2_2012
MK953230.1_RVB_Pig-wt_ESP_B333_2017_GXP4
MK953233.1_RVB_Pig-wt_ESP_B422_2017_GXP4
RVB_Pig-wt_ZAF_CAW50_2019
KR052709.1_RVB_Pig-tc_USA_LS00011_Ohio_XXXX_GXPX_A8
KX362384.1_RVB_Pig-wt_VNM_14151_62
MK953234.1_RVB_Pig-wt_ESP_P2B_2017_GXP4
MK953235.1_RVB_Pig-wt_ESP_VC20B_2017_GXP4
KX362395.1_RVB_Pig-wt_VNM_14176_8
KX362406.1_RVB_Pig-wt_VNM_14177_18
USA
MK953231.1_VB_Pig-wt_ESP_B377_2017_GXPX
MK953232.1_RVB_Pig-wt_ESP_B378_2017_GXPX
MK953236.1_RVB_Pig-wt_ESP_P1C_2017_GXP4
A8
L09722.1_RVB_Rat-wt_USA_IDIR_XXXX_G1P1
0.6
NSP2
N2
KX362374.1_RVB_Pig-wt_VNM_12089_7
AB673215.1_RVB_Pig-wt_JPN_PB-S22-3_2002_N8
AB673217.1_RVB_Pig-wt_JPN_PB-S40-1_2003_N8
AB673216.1_RVB_Pig-wt_JPN_PB-S26-1_2002_N9
KR052710.1_RVB_Pig-tc_USA_LS00011_Ohio_XXXX_GXPX_N10
MG271986.1_RVB_Pig-wt_USA_KS2_2012
KJ613656.1_RVB_Pig-wt_IND_AN142127_2013_G20PX
MK953223.1_RVB_Pig-wt_ESP_B377_2017_GXPX
MK953224.1_RVB_Pig-wt_ESP_B378_2017_GXPX
MK953228.1_RVB_Pig-wt_ESP_P1C_2017_GXP4
KX362407.1_RVB_Pig-wt_VNM_14177_18
RVB_Pig-wt_ZAF_CAW50_2019
KX362429.1_RVB_Pig-wt_VNM_14254_5_N10
KX362440.1_RVB_Pig-wt_VNM_14254_6
KX362385.1_RVB_Pig-wt_VNM_14151_62
MK953221.1_RVB_Pig-wt_ESP_B304_2017_G12PX
MK953222.1_RVB_Pig-wt_ESP_B333_2017_GXP4
MK953225.1_RVB_Pig-wt_ESP_B422_2017_GXP4
MK953226.1_RVB_Pig-wt_ESP_P2B_2017_GXP4
MG271987.1_RVB_Pig-wt_USA_KS1_2012
MK953227.1_RVB_Pig-wt_ESP_VC20B_2017_GXP4
KJ613653.1_RVB_Pig-wt_IND_NIV-094317_2009_GXPX_N10
KJ613657.1_RVB_Pig-wt_IND_AN142129_2013_G19PX
KJ613654.1_RVB_Pig-wt_IND_NIV-094327_2009_GXPX
KJ613655.1_RVB_Pig-wt_IND_NIV-094330_2009_G20PX
MG271988.1_RVB_Pig-wt_USA_IL3_2012
MG271992.1_RVB_Pig-wt_USA_IL12_2013
MG271991.1_RVB_Pig-wt_USA_IL7_2012
MG271990.1_RVB_Pig-wt_USA_IL5_2012
MG271993.1_RVB_Pig-wt_USA_IL17_2013
MG271989.1_RVB_Pig-wt_USA_IL4_2012_N10
N10
N3
N4
N5
AB673230.1_RVB_Pig-wt_JPN_PB-91-Z4_2008_N6
0.2

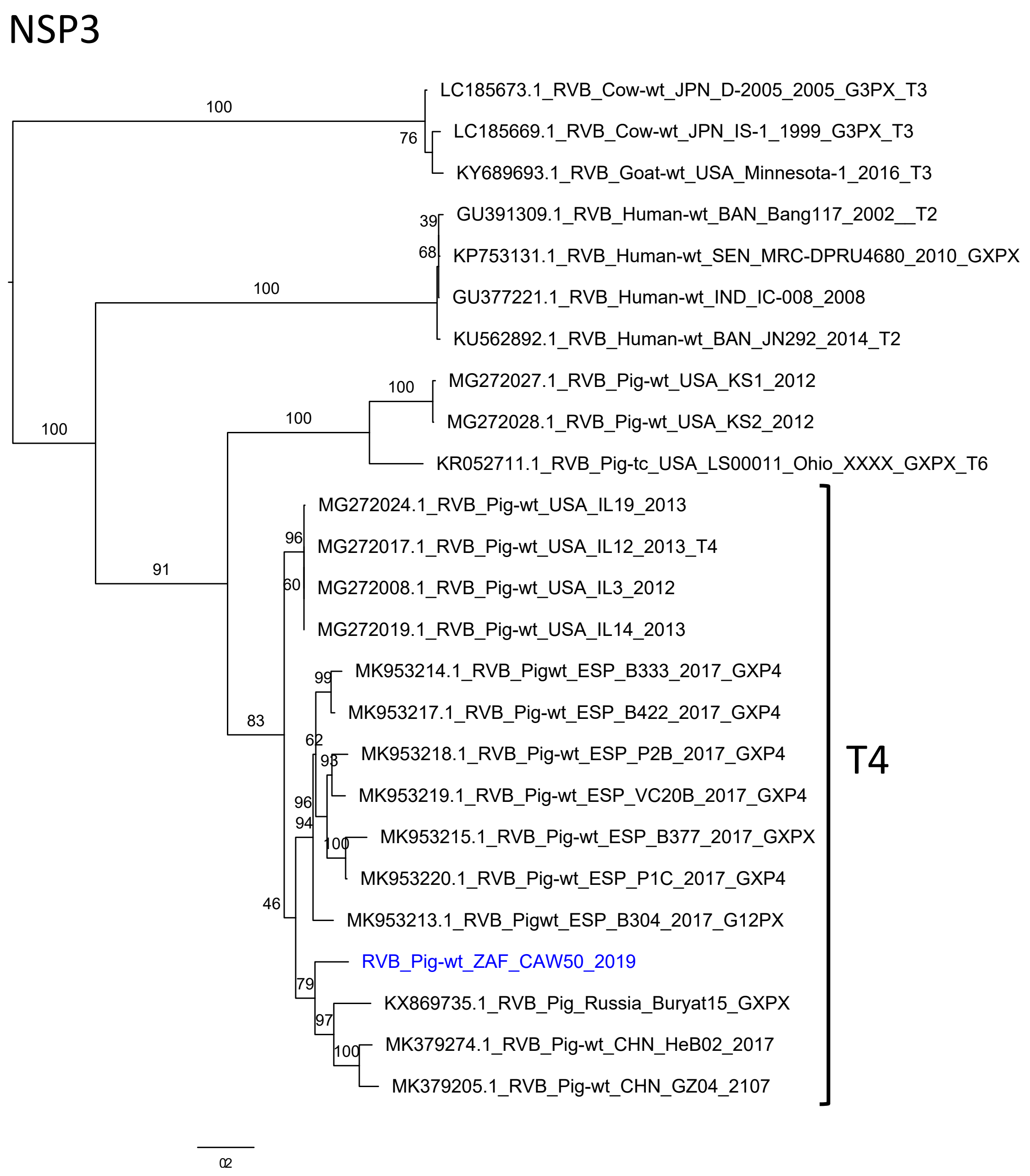
NSP3
LC185673.1_RVB_Cow-wt_JPN_D-2005_2005_G3PX_T3
LC185669.1_RVB_Cow-wt_JPN_IS-1_1999_G3PX_T3
KY689693.1_RVB_Goat-wt_USA_Minnesota-1_2016_T3
GU391309.1_RVB_Human-wt_BAN_Bang117_2002__T2
KP753131.1_RVB_Human-wt_SEN_MRC-DPRU4680_2010_GXPX
GU377221.1_RVB_Human-wt_IND_IC-008_2008
KU562892.1_RVB_Human-wt_BAN_JN292_2014_T2
MG272027.1_RVB_Pig-wt_USA_KS1_2012
MG272028.1_RVB_Pig-wt_USA_KS2_2012
KR052711.1_RVB_Pig-tc_USA_LS00011_Ohio_XXXX_GXPX_T6
MG272024.1_RVB_Pig-wt_USA_IL19_2013
MG272017.1_RVB_Pig-wt_USA_IL12_2013_T4
MG272008.1_RVB_Pig-wt_USA_IL3_2012
MG272019.1_RVB_Pig-wt_USA_IL14_2013
MK953214.1_RVB_Pigwt_ESP_B333_2017_GXP4
MK953217.1_RVB_Pig-wt_ESP_B422_2017_GXP4
MK953218.1_RVB_Pig-wt_ESP_P2B_2017_GXP4
MK953219.1_RVB_Pig-wt_ESP_VC20B_2017_GXP4
MK953215.1_RVB_Pig-wt_ESP_B377_2017_GXPX
MK953220.1_RVB_Pig-wt_ESP_P1C_2017_GXP4
MK953213.1_RVB_Pigwt_ESP_B304_2017_G12PX
RVB_Pig-wt_ZAF_CAW50_2019
KX869735.1_RVB_Pig_Russia_Buryat15_GXPX
MK379274.1_RVB_Pig-wt_CHN_HeB02_2017
MK379205.1_RVB_Pig-wt_CHN_GZ04_2107
T4
0.2

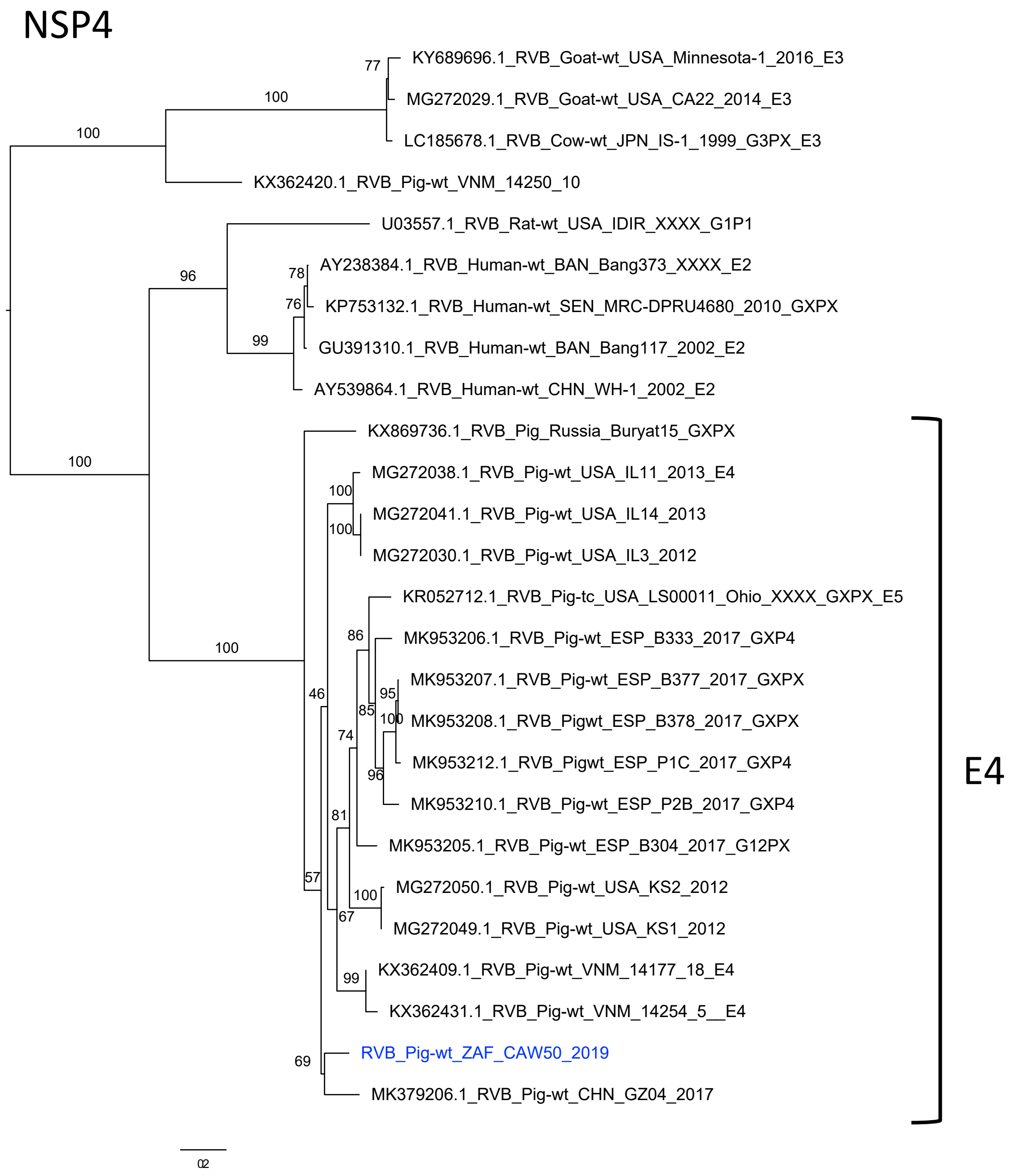
NSP4
KY689696.1_RVB_Goat-wt_USA_Minnesota-1_2016_E3
MG272029.1_RVB_Goat-wt_USA_CA22_2014_E3
LC185678.1_RVB_Cow-wt_JPN_IS-1_1999_G3PX_E3
KX362420.1_RVB_Pig-wt_VNM_14250_10
U03557.1_RVB_Rat-wt_USA_IDIR_XXXX_G1P1
AY238384.1_RVB_Human-wt_BAN_Bang373_XXXX_E2
KP753132.1_RVB_Human-wt_SEN_MRC-DPRU4680_2010_GXPX
GU391310.1_RVB_Human-wt_BAN_Bang117_2002_E2
AY539864.1_RVB_Human-wt_CHN_WH-1_2002_E2
KX869736.1_RVB_Pig_Russia_Buryat15_GXPX
MG272038.1_RVB_Pig-wt_USA_IL11_2013_E4
MG272041.1_RVB_Pig-wt_USA_IL14_2013
MG272030.1_RVB_Pig-wt_USA_IL3_2012
KR052712.1_RVB_Pig-tc_USA_LS00011_Ohio_XXXX_GXPX_E5
MK953206.1_RVB_Pig-wt_ESP_B333_2017_GXP4
MK953207.1_RVB_Pig-wt_ESP_B377_2017_GXPX
MK953208.1_RVB_Pigwt_ESP_B378_2017_GXPX
MK953212.1_RVB_Pigwt_ESP_P1C_2017_GXP4
MK953210.1_RVB_Pig-wt_ESP_P2B_2017_GXP4
MK953205.1_RVB_Pig-wt_ESP_B304_2017_G12PX
MG272050.1_RVB_Pig-wt_USA_KS2_2012
MG272049.1_RVB_Pig-wt_USA_KS1_2012
KX362409.1_RVB_Pig-wt_VNM_14177_18_E4
KX362431.1_RVB_Pig-wt_VNM_14254_5__E4
RVB_Pig-wt_ZAF_CAW50_2019
MK379206.1_RVB_Pig-wt_CHN_GZ04_2017
E4
0.2

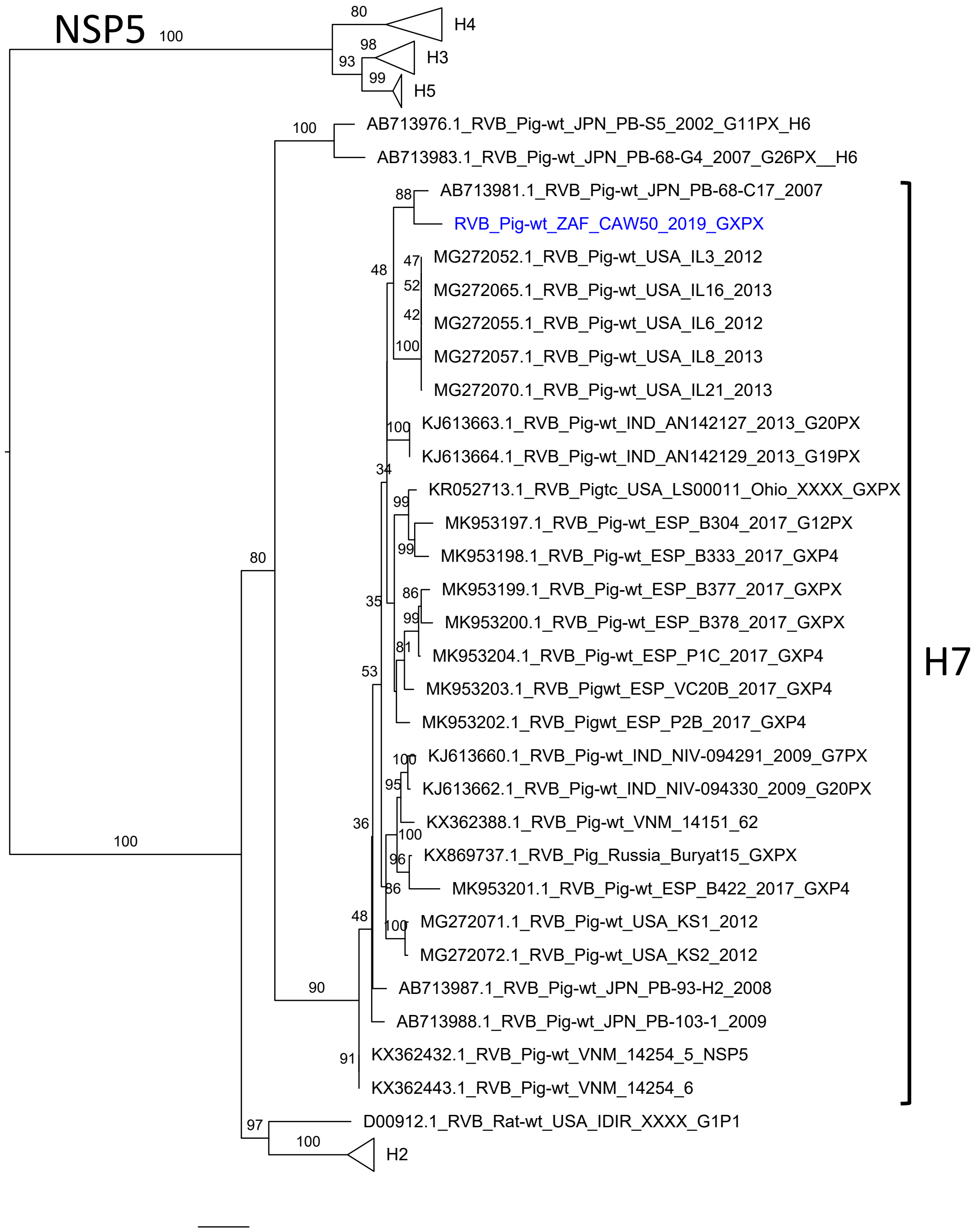

Figure S2. Rotavirus B Phylogenetic Analyses. The South African study strains in the phylogenetic trees are indicated in blue and other South African strains are indicated in green. Each gene was compared with sequences available in GenBank and nucleotide alignments were constructed using the MUSCLE algorithm in the MEGA X [35]. Phylogenetic trees were generated using IQtree implementing the Maximum Likelihood method, with ModelFinder and the trees were statistically supported using 1000 ultrafast bootstrap runs. For VP7: GTR+G4, VP1: GTR+G4; VP2: TIM2+G4, VP3: GTR+G4, NSP1: GTR+G4 and NSP2: GTR+G4; VP6: GTR+G4 VP4: TIM3+G4; NSP3: TIM+G4 and NSP4 TIM3+G4; NSP5: GTR+G4. The trees are drawn to scale, with branch lengths in the same units as those of the evolutionary distances used to infer the phylogenetic tree.
