# Supplementary material for: Phylogenetic Analyses of Rotavirus A, B and C Detected on a Porcine Farm in South Africa
Source: Viruses. 2024 Jun 8;16(6):934. doi: 10.3390/v16060934 (PMC11209240; doi:10.3390/v16060934)
Supplement: Supplementary file 1 [file viruses-16-00934-s001.zip › Suppl Docs Strydom et al/Figure S3_RVC Trees.pdf]

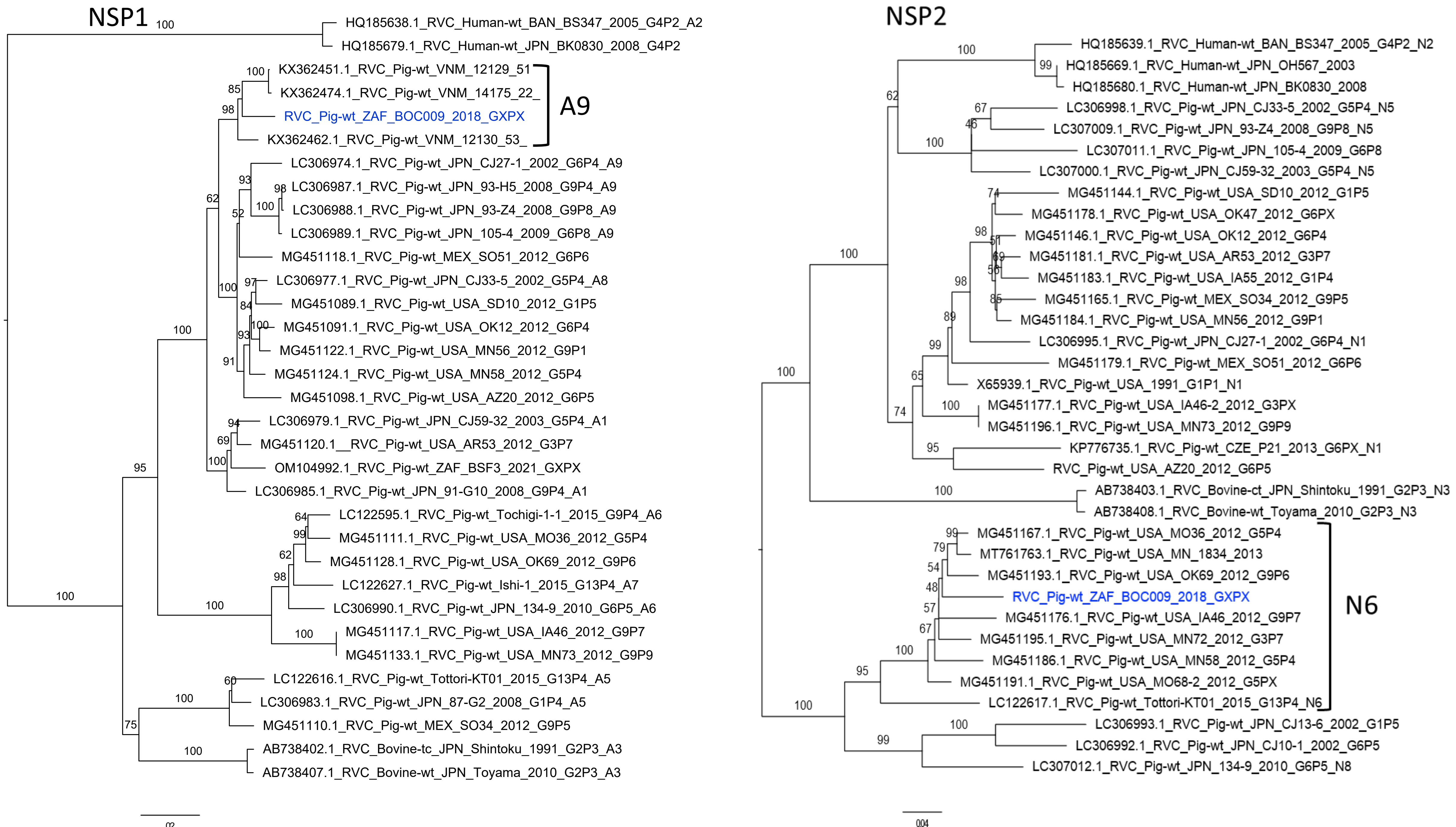
NSP1
HQ185638.1_RVC_Human-wt_BAN_BS347_2005_G4P2_A2
HQ185679.1_RVC_Human-wt_JPN_BK0830_2008_G4P2
KX362451.1_RVC_Pig-wt_VNM_12129_51
KX362474.1_RVC_Pig-wt_VNM_14175_22_
RVC_Pig-wt_ZAF_BOC009_2018_GXPX
KX362462.1_RVC_Pig-wt_VNM_12130_53_
A9
LC306974.1_RVC_Pig-wt_JPN_CJ27-1_2002_G6P4_A9
LC306987.1_RVC_Pig-wt_JPN_93-H5_2008_G9P4_A9
LC306988.1_RVC_Pig-wt_JPN_93-Z4_2008_G9P8_A9
LC306989.1_RVC_Pig-wt_JPN_105-4_2009_G6P8_A9
MG451118.1_RVC_Pig-wt_MEX_SO51_2012_G6P6
LC306977.1_RVC_Pig-wt_JPN_CJ33-5_2002_G5P4_A8
MG451089.1_RVC_Pig-wt_USA_SD10_2012_G1P5
MG451091.1_RVC_Pig-wt_USA_OK12_2012_G6P4
MG451122.1_RVC_Pig-wt_USA_MN56_2012_G9P1
MG451124.1_RVC_Pig-wt_USA_MN58_2012_G5P4
MG451098.1_RVC_Pig-wt_USA_AZ20_2012_G6P5
LC306979.1_RVC_Pig-wt_JPN_CJ59-32_2003_G5P4_A1
MG451120.1__RVC_Pig-wt_USA_AR53_2012_G3P7
OM104992.1_RVC_Pig-wt_ZAF_BSF3_2021_GXPX
LC306985.1_RVC_Pig-wt_JPN_91-G10_2008_G9P4_A1
LC122595.1_RVC_Pig-wt_Tochigi-1-1_2015_G9P4_A6
MG451111.1_RVC_Pig-wt_USA_MO36_2012_G5P4
MG451128.1_RVC_Pig-wt_USA_OK69_2012_G9P6
LC122627.1_RVC_Pig-wt_Ishi-1_2015_G13P4_A7
LC306990.1_RVC_Pig-wt_JPN_134-9_2010_G6P5_A6
MG451117.1_RVC_Pig-wt_USA_IA46_2012_G9P7
MG451133.1_RVC_Pig-wt_USA_MN73_2012_G9P9
LC122616.1_RVC_Pig-wt_Tottori-KT01_2015_G13P4_A5
LC306983.1_RVC_Pig-wt_JPN_87-G2_2008_G1P4_A5
MG451110.1_RVC_Pig-wt_MEX_SO34_2012_G9P5
AB738402.1_RVC_Bovine-tc_JPN_Shintoku_1991_G2P3_A3
AB738407.1_RVC_Bovine-wt_JPN_Toyama_2010_G2P3_A3
NSP2
HQ185639.1_RVC_Human-wt_BAN_BS347_2005_G4P2_N2
HQ185669.1_RVC_Human-wt_JPN_OH567_2003
HQ185680.1_RVC_Human-wt_JPN_BK0830_2008
LC306998.1_RVC_Pig-wt_JPN_CJ33-5_2002_G5P4_N5
LC307009.1_RVC_Pig-wt_JPN_93-Z4_2008_G9P8_N5
LC307011.1_RVC_Pig-wt_JPN_105-4_2009_G6P8
LC307000.1_RVC_Pig-wt_JPN_CJ59-32_2003_G5P4_N5
MG451144.1_RVC_Pig-wt_USA_SD10_2012_G1P5
MG451178.1_RVC_Pig-wt_USA_OK47_2012_G6PX
MG451146.1_RVC_Pig-wt_USA_OK12_2012_G6P4
MG451181.1_RVC_Pig-wt_USA_AR53_2012_G3P7
MG451183.1_RVC_Pig-wt_USA_IA55_2012_G1P4
MG451165.1_RVC_Pig-wt_MEX_SO34_2012_G9P5
MG451184.1_RVC_Pig-wt_USA_MN56_2012_G9P1
LC306995.1_RVC_Pig-wt_JPN_CJ27-1_2002_G6P4_N1
MG451179.1_RVC_Pig-wt_MEX_SO51_2012_G6P6
X65939.1_RVC_Pig-wt_USA_1991_G1P1_N1
MG451177.1_RVC_Pig-wt_USA_IA46-2_2012_G3PX
MG451196.1_RVC_Pig-wt_USA_MN73_2012_G9P9
KP776735.1_RVC_Pig-wt_CZE_P21_2013_G6PX_N1
RVC_Pig-wt_USA_AZ20_2012_G6P5
AB738403.1_RVC_Bovine-ct_JPN_Shintoku_1991_G2P3_N3
AB738408.1_RVC_Bovine-wt_Toyama_2010_G2P3_N3
MG451167.1_RVC_Pig-wt_USA_MO36_2012_G5P4
MT761763.1_RVC_Pig-wt_USA_MN_1834_2013
MG451193.1_RVC_Pig-wt_USA_OK69_2012_G9P6
RVC_Pig-wt_ZAF_BOC009_2018_GXPX
MG451176.1_RVC_Pig-wt_USA_IA46_2012_G9P7
MG451195.1_RVC_Pig-wt_USA_MN72_2012_G3P7
MG451186.1_RVC_Pig-wt_USA_MN58_2012_G5P4
MG451191.1_RVC_Pig-wt_USA_MO68-2_2012_G5PX
LC122617.1_RVC_Pig-wt_Tottori-KT01_2015_G13P4_N6
N6
LC306993.1_RVC_Pig-wt_JPN_CJ13-6_2002_G1P5
LC306992.1_RVC_Pig-wt_JPN_CJ10-1_2002_G6P5
LC307012.1_RVC_Pig-wt_JPN_134-9_2010_G6P5_N8

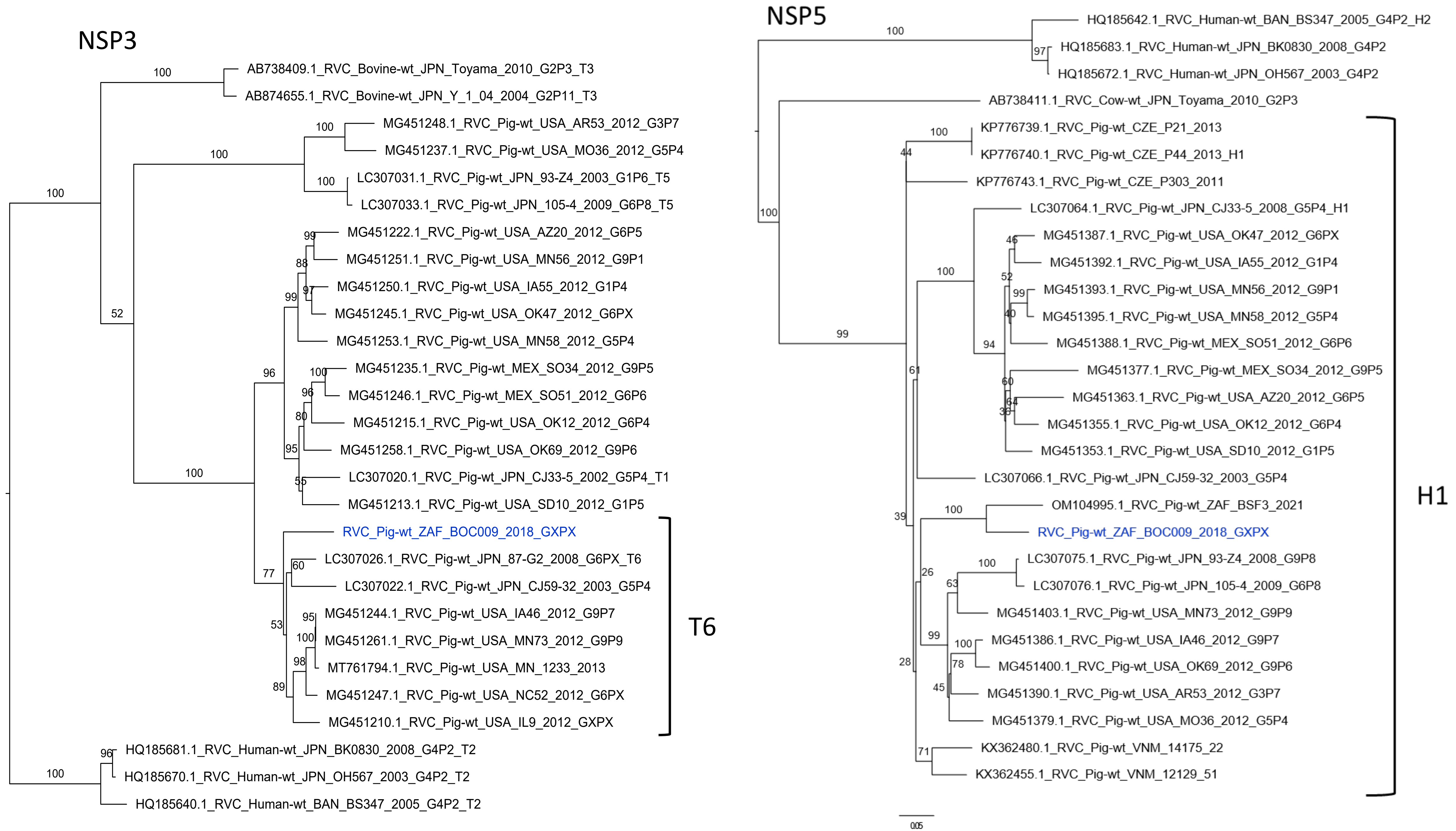
NSP3
AB738409.1_RVC_Bovine-wt_JPN_Toyama_2010_G2P3_T3
AB874655.1_RVC_Bovine-wt_JPN_Y_1_04_2004_G2P11_T3
MG451248.1_RVC_Pig-wt_USA_AR53_2012_G3P7
MG451237.1_RVC_Pig-wt_USA_MO36_2012_G5P4
LC307031.1_RVC_Pig-wt_JPN_93-Z4_2003_G1P6_T5
LC307033.1_RVC_Pig-wt_JPN_105-4_2009_G6P8_T5
MG451222.1_RVC_Pig-wt_USA_AZ20_2012_G6P5
MG451251.1_RVC_Pig-wt_USA_MN56_2012_G9P1
MG451250.1_RVC_Pig-wt_USA_IA55_2012_G1P4
MG451245.1_RVC_Pig-wt_USA_OK47_2012_G6PX
MG451253.1_RVC_Pig-wt_USA_MN58_2012_G5P4
MG451235.1_RVC_Pig-wt_MEX_SO34_2012_G9P5
MG451246.1_RVC_Pig-wt_MEX_SO51_2012_G6P6
MG451215.1_RVC_Pig-wt_USA_OK12_2012_G6P4
MG451258.1_RVC_Pig-wt_USA_OK69_2012_G9P6
LC307020.1_RVC_Pig-wt_JPN_CJ33-5_2002_G5P4_T1
MG451213.1_RVC_Pig-wt_USA_SD10_2012_G1P5
RVC_Pig-wt_ZAF_BOC009_2018_GXPX
LC307026.1_RVC_Pig-wt_JPN_87-G2_2008_G6PX_T6
LC307022.1_RVC_Pig-wt_JPN_CJ59-32_2003_G5P4
MG451244.1_RVC_Pig-wt_USA_IA46_2012_G9P7
MG451261.1_RVC_Pig-wt_USA_MN73_2012_G9P9
MT761794.1_RVC_Pig-wt_USA_MN_1233_2013
MG451247.1_RVC_Pig-wt_USA_NC52_2012_G6PX
MG451210.1_RVC_Pig-wt_USA_IL9_2012_GXPX
T6
HQ185681.1_RVC_Human-wt_JPN_BK0830_2008_G4P2_T2
HQ185670.1_RVC_Human-wt_JPN_OH567_2003_G4P2_T2
HQ185640.1_RVC_Human-wt_BAN_BS347_2005_G4P2_T2
NSP5
HQ185642.1_RVC_Human-wt_BAN_BS347_2005_G4P2_H2
HQ185683.1_RVC_Human-wt_JPN_BK0830_2008_G4P2
HQ185672.1_RVC_Human-wt_JPN_OH567_2003_G4P2
AB738411.1_RVC_Cow-wt_JPN_Toyama_2010_G2P3
KP776739.1_RVC_Pig-wt_CZE_P21_2013
KP776740.1_RVC_Pig-wt_CZE_P44_2013_H1
KP776743.1_RVC_Pig-wt_CZE_P303_2011
LC307064.1_RVC_Pig-wt_JPN_CJ33-5_2008_G5P4_H1
MG451387.1_RVC_Pig-wt_USA_OK47_2012_G6PX
MG451392.1_RVC_Pig-wt_USA_IA55_2012_G1P4
MG451393.1_RVC_Pig-wt_USA_MN56_2012_G9P1
MG451395.1_RVC_Pig-wt_USA_MN58_2012_G5P4
MG451388.1_RVC_Pig-wt_MEX_SO51_2012_G6P6
MG451377.1_RVC_Pig-wt_MEX_SO34_2012_G9P5
MG451363.1_RVC_Pig-wt_USA_AZ20_2012_G6P5
MG451355.1_RVC_Pig-wt_USA_OK12_2012_G6P4
MG451353.1_RVC_Pig-wt_USA_SD10_2012_G1P5
LC307066.1_RVC_Pig-wt_JPN_CJ59-32_2003_G5P4
OM104995.1_RVC_Pig-wt_ZAF_BSF3_2021
RVC_Pig-wt_ZAF_BOC009_2018_GXPX
LC307075.1_RVC_Pig-wt_JPN_93-Z4_2008_G9P8
LC307076.1_RVC_Pig-wt_JPN_105-4_2009_G6P8
MG451403.1_RVC_Pig-wt_USA_MN73_2012_G9P9
MG451386.1_RVC_Pig-wt_USA_IA46_2012_G9P7
MG451400.1_RVC_Pig-wt_USA_OK69_2012_G9P6
MG451390.1_RVC_Pig-wt_USA_AR53_2012_G3P7
MG451379.1_RVC_Pig-wt_USA_MO36_2012_G5P4
KX362480.1_RVC_Pig-wt_VNM_14175_22
KX362455.1_RVC_Pig-wt_VNM_12129_51
H1
0.05

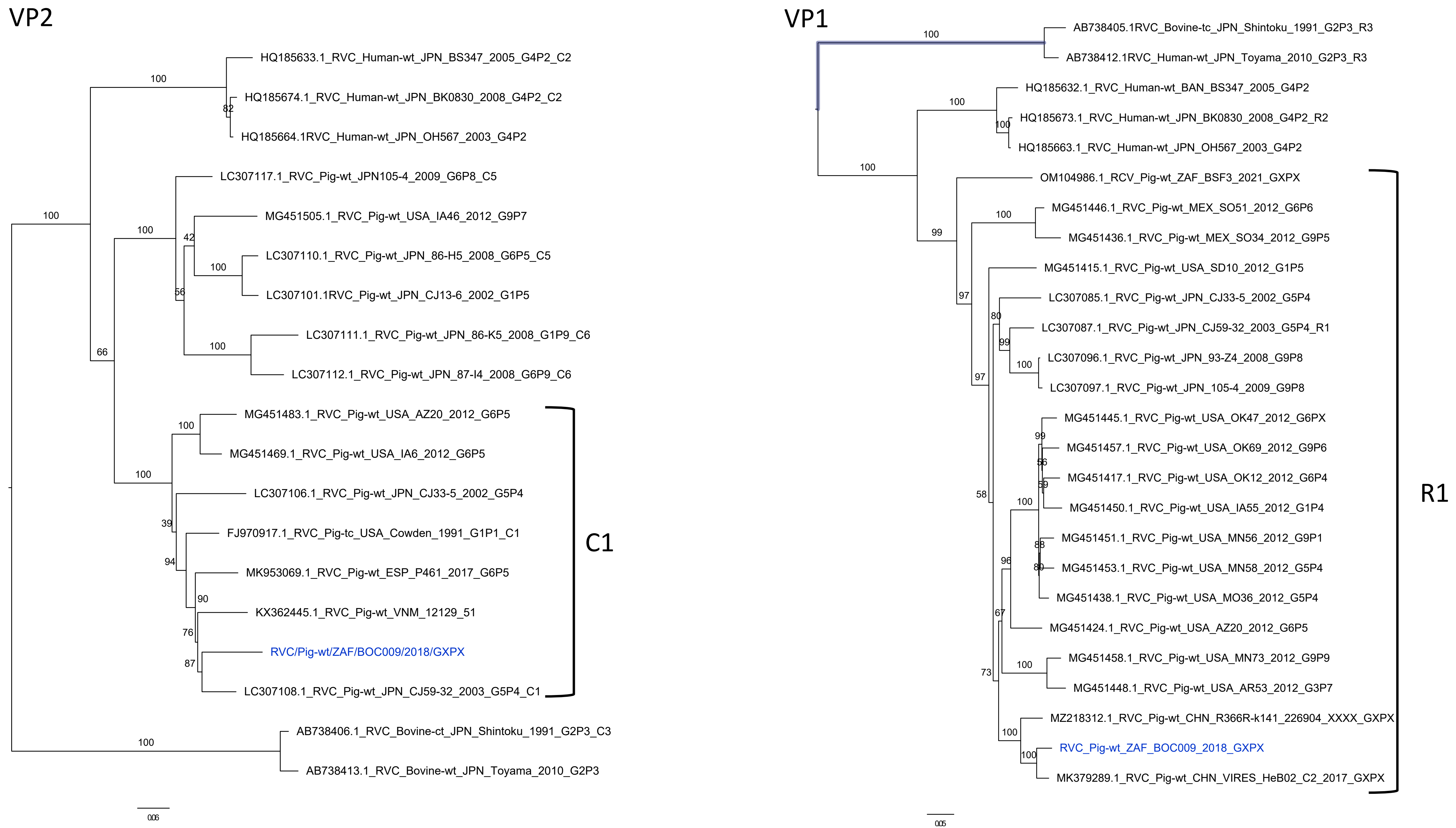
VP2
HQ185633.1_RVC_Human-wt_JPN_BS347_2005_G4P2_C2
HQ185674.1_RVC_Human-wt_JPN_BK0830_2008_G4P2_C2
HQ185664.1RVC_Human-wt_JPN_OH567_2003_G4P2
LC307117.1_RVC_Pig-wt_JPN105-4_2009_G6P8_C5
MG451505.1_RVC_Pig-wt_USA_IA46_2012_G9P7
LC307110.1_RVC_Pig-wt_JPN_86-H5_2008_G6P5_C5
LC307101.1RVC_Pig-wt_JPN_CJ13-6_2002_G1P5
LC307111.1_RVC_Pig-wt_JPN_86-K5_2008_G1P9_C6
LC307112.1_RVC_Pig-wt_JPN_87-I4_2008_G6P9_C6
MG451483.1_RVC_Pig-wt_USA_AZ20_2012_G6P5
MG451469.1_RVC_Pig-wt_USA_IA6_2012_G6P5
LC307106.1_RVC_Pig-wt_JPN_CJ33-5_2002_G5P4
FJ970917.1_RVC_Pig-tc_USA_Cowden_1991_G1P1_C1
MK953069.1_RVC_Pig-wt_ESP_P461_2017_G6P5
KX362445.1_RVC_Pig-wt_VNM_12129_51
RVC/Pig-wt/ZAF/BOC009/2018/GXPX
LC307108.1_RVC_Pig-wt_JPN_CJ59-32_2003_G5P4_C1
AB738406.1_RVC_Bovine-ct_JPN_Shintoku_1991_G2P3_C3
AB738413.1_RVC_Bovine-wt_JPN_Toyama_2010_G2P3
C1
0.05
VP1
AB738405.1RVC_Bovine-tc_JPN_Shintoku_1991_G2P3_R3
AB738412.1RVC_Human-wt_JPN_Toyama_2010_G2P3_R3
HQ185632.1_RVC_Human-wt_BAN_BS347_2005_G4P2
HQ185673.1_RVC_Human-wt_JPN_BK0830_2008_G4P2_R2
HQ185663.1_RVC_Human-wt_JPN_OH567_2003_G4P2
OM104986.1_RCV_Pig-wt_ZAF_BSF3_2021_GXPX
MG451446.1_RVC_Pig-wt_MEX_SO51_2012_G6P6
MG451436.1_RVC_Pig-wt_MEX_SO34_2012_G9P5
MG451415.1_RVC_Pig-wt_USA_SD10_2012_G1P5
LC307085.1_RVC_Pig-wt_JPN_CJ33-5_2002_G5P4
LC307087.1_RVC_Pig-wt_JPN_CJ59-32_2003_G5P4_R1
LC307096.1_RVC_Pig-wt_JPN_93-Z4_2008_G9P8
LC307097.1_RVC_Pig-wt_JPN_105-4_2009_G9P8
MG451445.1_RVC_Pig-wt_USA_OK47_2012_G6PX
MG451457.1_RVC_Pig-wt_USA_OK69_2012_G9P6
MG451417.1_RVC_Pig-wt_USA_OK12_2012_G6P4
MG451450.1_RVC_Pig-wt_USA_IA55_2012_G1P4
MG451451.1_RVC_Pig-wt_USA_MN56_2012_G9P1
MG451453.1_RVC_Pig-wt_USA_MN58_2012_G5P4
MG451438.1_RVC_Pig-wt_USA_MO36_2012_G5P4
MG451424.1_RVC_Pig-wt_USA_AZ20_2012_G6P5
MG451458.1_RVC_Pig-wt_USA_MN73_2012_G9P9
MG451448.1_RVC_Pig-wt_USA_AR53_2012_G3P7
MZ218312.1_RVC_Pig-wt_CHN_R366R-k141_226904_XXXX_GXPX
RVC_Pig-wt_ZAF_BOC009_2018_GXPX
MK379289.1_RVC_Pig-wt_CHN_VIRES_HeB02_C2_2017_GXPX
R1
0.05

Figure S3. Rotavirus C Phylogenetic Analyses. The South African study strains in the phylogenetic trees are indicated in blue and other South African strains are indicated in green. Each gene was compared with sequences available in GenBank and nucleotide alignments were constructed using the MUSCLE algorithm in the MEGA X [35]. Phylogenetic trees were generated using IQtree implementing the Maximum Likelihood method, with ModelFinder and the trees were statistically supported using 1000 ultrafast bootstrap runs. For VP7: TIM2+G4, VP1: GTR+G4; VP2: GTR+G4, NSP1: GTR+G4 and NSP2: TIM2+G4; VP4: GTR+G4; NSP3: TN+G4 and NSP4: TPM3+G4; NSP5: TIM2+G4. The trees are drawn to scale, with branch lengths in the same units as those of the evolutionary distances used to infer the phylogenetic tree.
