# Supplementary material for: Phylogenetic Analyses of Rotavirus A, B and C Detected on a Porcine Farm in South Africa
Source: Viruses. 2024 Jun 8;16(6):934. doi: 10.3390/v16060934 (PMC11209240; doi:10.3390/v16060934)
Supplement: Supplementary file 1 [file viruses-16-00934-s001.zip › Suppl Docs Strydom et al/Table S1.docx]

Table S1: Sequences used for reference mapping

| **Rotavirus** | **Genome Segment (GS)** | **Accession Number** |
| --- | --- | --- |
| RVA | GS1 – VP1 | AB924084.1 |
|  | GS2 – VP2 | AB924085.1 |
|  | GS3 – VP3 | AB924086.1 |
|  | GS4 – VP4 (P[6]) | KX363402.1 |
|  | GS4 – VP4 (P[13]) | MH267274.1, KX988278.1 |
|  | GS4 – VP4 (P[23]) | JF781161.1 |
|  | GS5 – NSP1 | MT784838.1 |
|  | GS6 – VP6 | KF500200.1 |
|  | GS7 – NSP3 | KR632628.1 |
|  | GS8 – NSP2 | JQ309142.1 |
|  | GS9 – VP7 | KP752772.1 |
|  | GS10 – NSP4 | MT784862.1 |
|  | GS11 – NSP5 | AB741659.1 |
| RVB | GS1 – VP1 | KX362389.1 |
|  | GS2 – VP2 | KR052715.1, MG272106.1 |
|  | GS3 – VP3 | MG272126.1 |
|  | GS4 – VP4 | MG272151.1 |
|  | GS5 – NSP1 | KX869733.1 |
|  | GS6 – VP6 | MG272174.1 |
|  | GS7 – NSP3 | KX869735.1 |
|  | GS8 – NSP2 | AB673231.1 |
|  | GS9 – VP7 | MF522263.1, MG272196.1 |
|  | GS10 – NSP4 | MK379321.1 |
|  | GS11 – NSP5 | KX869737.1 |
| RVC | GS1 – VP1 | MN809644.1 |
|  | GS2 – VP2 | KP982880.1 |
|  | GS3 – VP3 | MN809635.1, MN809646.1 |
|  | GS4 – VP4 | KC164679.1 |
|  | GS5 – NSP1 | KY909951.1 |
|  | GS6 – VP6 | KC164674.1, KC164677.1 |
|  | GS7 – NSP3 | LC307026.1 |
|  | GS8 – NSP2 | LC307005.1 |
|  | GS9 – VP7 | AB905222.1 |
|  | GS10 – NSP4 | KX373864.1 |
|  | GS11 – NSP5 | KP776743.1 |
